# Isolation of C1 through C4 derivatives from CO using heteroleptic uranium(iii) metallocene aryloxide complexes†

**DOI:** 10.1039/d2sc06375a

**Published:** 2023-02-06

**Authors:** Robert J. Ward, Iker del Rosal, Steven P. Kelley, Laurent Maron, Justin R. Walensky

**Affiliations:** a Department of Chemistry, University of Missouri Columbia MO 65211 USA walenskyj@missouri.edu; b Universite de Toulouse, CNRS, INSA, UMR UMR 5215 LPCNO 135 Avenue de Ranguiel 31077 Toulouse France

## Abstract

The conversion of C1 feedstock molecules such as CO into commodity chemicals is a desirable, but challenging, endeavour. When the U(iii) complex, [(C_5_Me_5_)_2_U(O-2,6-^*t*^Bu_2_-4-MeC_6_H_2_)], is exposed to 1 atm of CO, only coordination is observed by IR spectroscopy as well as X-ray crystallography, unveiling a rare structurally characterized f element carbonyl. However, using [(C_5_Me_5_)_2_(MesO)U (THF)], Mes = 2,4,6-Me_3_C_6_H_2_, reaction with CO forms the bridging ethynediolate species, [{(C_5_Me_5_)_2_(MesO)U}_2_(μ_2_-OCCO)]. While ethynediolate complexes are known, their reactivity has not been reported in much detail to afford further functionalization. For example, addition of more CO to the ethynediolate complex with heating forms a ketene carboxylate, [{(C_5_Me_5_)_2_(MesO)U}_2_(*μ*_2_:*κ*^2^:*η*^1^-C_3_O_3_)], which can be further reacted with CO_2_ to yield a ketene dicarboxylate complex, [{(C_5_Me_5_)_2_(MesO)U}_2_(*μ*_2_:*κ*^2^:*κ*^2^-C_4_O_5_)]. Since the ethynediolate showed reactivity with more CO, we explored its reactivity further. A [2 + 2] cycloaddition is observed with diphenylketene to yield [{(C_5_Me_5_)_2_U}_2_(OC(CPh_2_)C(=O)CO)] with concomitant formation of [(C_5_Me_5_)_2_U(OMes)_2_]. Surprisingly, reaction with SO_2_ shows rare S–O bond cleavage to yield the unusual [(O_2_CC(O)(SO)]^2−^ bridging ligand between two U(iv) centres. All complexes have been characterized using spectroscopic and structural methods, and the reaction of the ethynediolate with CO to form the ketene carboxylate has been investigated computationally as well as the reaction with SO_2_.

## Abstract 2

Functionalization of CO from C1 to C4 is acheived using a heteroleptic uranium(iii) complex.

## Graphical abstract

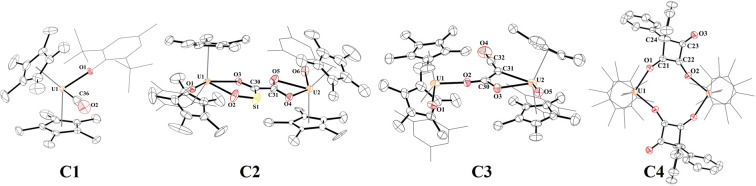

## Introduction

Due to the problems associated with our currently used hydrocarbon resources, there is great interest in the conversion of C1 feedstock molecules such as CO and CO_2_ into larger liquid hydrocarbons and other desirable commodity chemicals. This is well established using the Fischer–Tropsch process which converts syngas mixtures (H_2_/CO) into chain hydrocarbons using heterogeneous transition metal catalysts. However, Fischer–Tropsch chemistry remains underdeveloped with respect to homogeneous catalysis,^1–3^ and stoichiometric reactions involving metal complexes and CO can provide insight into CO functionalization,^4,5^ especially the coupling of CO molecules, by uncovering novel reactivity and moieties.

One method of functionalizing CO is through homologation.^6^ The reductive coupling of CO by molecular metal complexes has received attention in the development of strategies for utilizing C1 feedstock molecules for providing insight into heterogeneous reactions.^7,8^ Homologation of CO is difficult due to the high bond dissociation energy of the CO triple bond, rendering the molecule relatively inert compared to other small molecules. In addition, CO is the quintessential coordinating ligand for low-valent, electron-rich transition metal complexes, which are typically more susceptible to undergoing redox chemistry. Actinides are large, electropositive metals which do not possess the ability to back bond to CO in a similar manner to transition metals^9^ and uranium can span oxidation states of +1 to +6, giving a rich redox chemistry.^10–12^

The quest for CO reductive coupling reactions began in the 1800s with Liebig and Gmelin reporting that CO reacts with molten potassium to produce (C_5_O_5_)^2−^ and (C_6_O_6_)^2−^ dianions.^13,14^ Since then, other s-block^15–22^ as well as p-block^23–29^ complexes have been used for CO reduction over the years. There are only a handful of reports of CO reductive coupling with lanthanides. The Evans group reported that the reaction of [(C_5_Me_5_)_2_Sm(THF)_2_]^30^ or [{(C_5_Me_5_)_2_La}_2_(*μ*_2_:*η*^2^-N_2_)]^31^ with CO both produce a similar ketenecarboxylate product. In addition, Evans and co-workers have also observed CO radical, ethynediolate, or enediolate formation^32,33^ with Y(ii) or treatment of trivalent lanthanide with potassium graphite in their LnZ_3_/K reactivity. While carrying out this study, Nocton and co-workers demonstrated CO homologation with a thulium(ii) complex, [(1,2,4-^*t*^Bu_3_C_5_H_2_)_2_Tm], forming an ethynediolate which can then be further functionalized into a ketenecarboxylate with additional CO, followed by insertion of CO_2_ into the Tm–C bond to yield a ketenedicarboxylate.^34^

The reactivity of CO with uranium complexes is well established, but mainly with U(iv) complexes in which migratory insertion occurs into uranium-element bonds.^35–51^ These include examples in which CO inserts into a uranium-element bond, followed by C–C bond coupling with another CO molecule. The reductive coupling of CO has also been beautifully demonstrated by Cloke and co-workers by varying the steric properties of substituted-cyclooctatetrienyl, [C_8_H_6_(SiR_3_)_2_)]^2−^, R = ^*i*^Pr, Me, cyclopentadienyl, (C_5_Me_4_R)^1−^, R = Me, H, uranium(iii) complexes to obtain different reductively coupled CO products.^52–58^ The Arnold group was the next to observe the ethynediolate moiety with the reaction of [{(Me_3_Si)_2_N}_3_U] with CO. Heating the ethynediolate led to intramolecular C–H bond activation across the alkyne, forming a seven-membered metallocycle containing an enediolate.^59^ Additionally, Arnold reported a homoleptic U(iii) aryloxide complex, U(O-2,6-^*t*^Bu_2_C_6_H_3_)_3_,^60^ which upon reaction with CO also formed an ethynediolate.^61^ The Liddle group isolated an ethynediolate through the reaction of U(iii) ligated with the sterically encumbering Tren^DMSB^ ligand, [U(Tren^DMSB^)], Tren^DMSB^ = N(CH_2_CH_2_NSiMe_2_^*t*^Bu)_3_.^62^ Thermolysis of the ethynediolate at 80 °C resulted in the insertion of the ethynediolate into one of the N–Si bonds of the Tren^DMSB^ ligands with concomitant protonation of the ethynediolate and formation of an oxo-bridge between two uranium(iv) centers. The Meyer group has also observed a unique CO bridged complex, [{(L)U}_2_(*μ*_2_-CO)], *L* = 1,4,7-tris(3,5-di-*tert*-butyl-2-hydroxybenzylate-1,4,7-triazacyclononane.^63^ To our knowledge, the only report of further functionalization of a uranium ethynediolate complex, through addition of a substrate, is a structure in the Cambridge Crystallographic Database Centre, [{(2,6-^t^Bu_2_C_6_H_3_O)_3_U}_2_(*μ*_2_-OC(H)=C(BC_8_H_15_)O)], by Mansell and Arnold in which hydroboration occurs across the CC triple bond.^64^ Carbon monoxide functionalization with uranium nitrides has also been reported.^65–67^

Herein, we describe the use of two metallocene aryloxide uranium(iii) complexes, [(C_5_Me_5_)_2_(2,6-^*t*^Bu_2_-4-MeC_6_H_2_–O)U], 2,6-^*t*^Bu_2_-4-MeC_6_H_2_–O = BHT, and [(C_5_Me_5_)_2_(2,4,6-Me_3_C_6_H_2_–O)U(THF)], 2,4,6-Me_3_C_6_H_2_–O = Mes, and their reactivity with CO. In the former, BHT derivative, only coordination takes place as observed by IR spectroscopy and structural determination by X-ray crystallography. This is only the third structure of an f element carbonyl. However, upon reductive coupling of CO to form the ethynediolate, [{(C_5_Me_5_)_2_(MesO)U}_2_{*μ*_2_-(OCCO)}], a C_2_ moiety is found with the mesityl substituted aryloxide. Analogous to Nocton's recent findings, the ethynediolate can react with CO at elevated temperatures to yield the C_3_ ketenecarboxylate, which can react with CO_2_ to yield a C_4_ ketenedicarboxylate. In addition, reactions with diphenylketene and SO_2_ (DABSO) were investigated which showed [2 + 2] cycloaddition reactivity.

## Results and discussion

Uranium(iii) aryloxide starting materials have been previously reported from the reaction of [(C_5_Me_5_)_2_UI(THF)] with the appropriate potassium aryloxide salt, yielding [(C_5_Me_5_)_2_(2,6-^*t*^Bu_2_-4-MeC_6_H_2_–O)U], 1,^68^ and [(C_5_Me_5_)_2_(MesO)U(THF)], 2.^69^ Treatment of 1 atm of CO to 1 in hexamethyldisiloxane has a colour change from dark green to brown, eq 1. The liquid IR spectrum of the reaction showed a strong absorption at 1893 cm^−1^ indicating significant backbonding to the CO π* orbitals.^9^ With ^13^CO, a stretching frequency of 1853 cm^−1^ is observed, consistent with the reduced mass of CO *versus*^13^CO. Therefore, we formulated the new compound as [(C_5_Me_5_)_2_(2,6-^*t*^Bu_2_-4-MeC_6_H_2_–O)U(CO)], 3.1
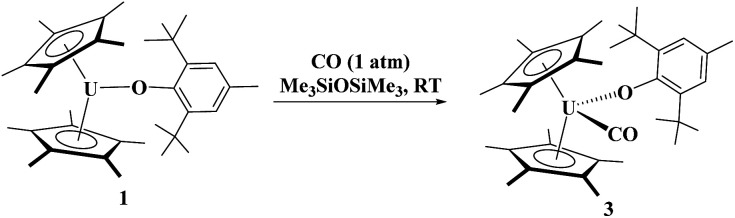


**Fig. 1:**
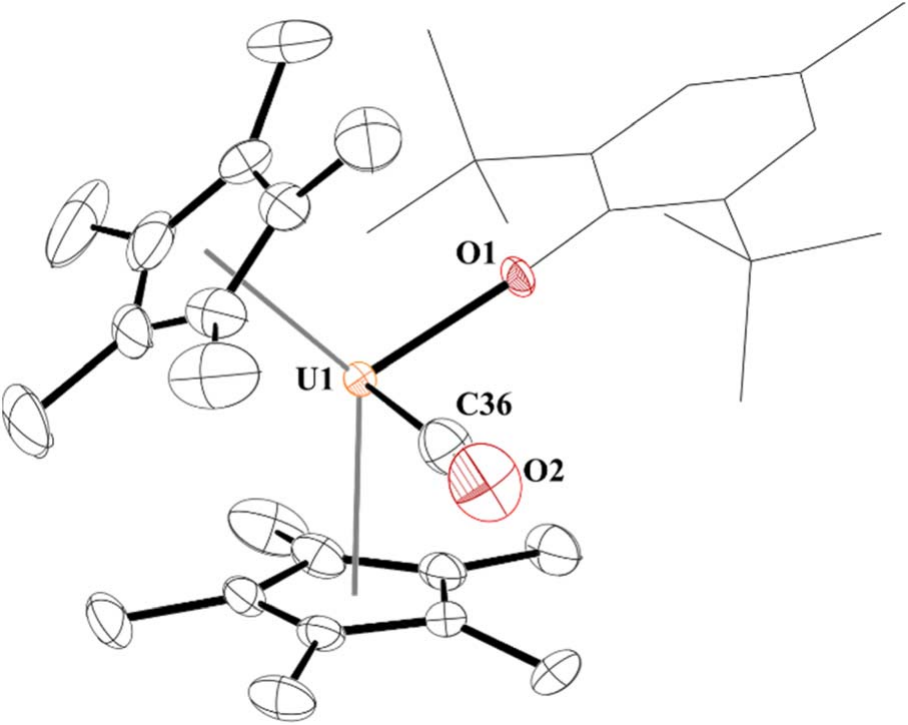
Thermal ellipsoid plot of 3 shown at the 50% probability level. The hydrogen atoms have been omitted and the (2,6-^*t*^Bu_2_-4-MeC_6_H_2_) group is shown in wireframe for clarity.

The ^1^H NMR spectrum of 3 consists of resonances at −3.75 ppm and 5.57 ppm for the *tert*-butyl and methyl groups, respectively, as well as the (C_5_Me_5_)^1−^ resonance at −5.61 ppm. When tabulating all the uranium carbonyl complexes reported, Table 1, we noticed that 3 has one of the lowest stretching frequencies reported and falls between the two structurally characterized complexes, [(C_5_Me_5_)_3_U(CO)], 3a, at 1922 cm^−1^ and [(C_5_Me_4_H)_3_U(CO)], 3b, at 1880 cm^−1^. Gratifyingly, brown crystals suitable for X-ray crystallographic analysis were grown from the reaction mixture, Fig. 1.

**Table 1:** CO stretching frequencies (cm^−1^) for all reported uranium complexes in the order of increasing backbonding

Compound	*ν* CO cm^−1^
CO^70^	2143 (g)
[(C_5_H_4_SiMe_3_)_3_U(CO)]^71^	1976 (C_5_H_12_)
	1969 (KBr)
[(C_5_Me_5_)_2_(As_2_Mes_2_)U(CO)]^72^	1939 (C_6_D_6_)
[(C_5_Me_5_)_3_U(CO)],^73^3a	1922 (KBr)
	1925 (C_6_H_6_)
[{C_8_H_6_(SiMe_3_)_2_}(C_5_Me_5_)U(CO)]^54^	1920 (*d*_8_-toluene)
[(C_5_Me_5_)_2_(2,6-^t^Bu_2_-4-MeC_6_H_2_)U(CO)], 3 (this work)	1904 (KBr)
	1893 (C_6_D_6_)
[(C_5_Me_4_H)_3_U(CO)],^74^3b	1880 (KBr)
	1900 (petrol. ether)

Complex 3 is stable in solution for several hours. Monitoring of the ^1^H NMR spectrum showed 60% conversion of 3 to 1 over the course of 15 hours. In addition, 3 is also stable in the solid-state with a stretching frequency of 1904 cm^−1^ (KBr). Under reduced pressure, complex 3 converts to 1 in ∼20 minutes.

Complex 3 is only the third structurally characterized carbonyl complex with an f element, Fig. 1. Complex 3 adopts a distorted tetrahedral geometry with a centroid–U–centroid angle of 132.60°, centroid–U–CO angles of 91.27 and 90.62°, and centroid-U-O(aryloxide) angles of 113.78 and 112.96°. These metrics are similar to 1, and for other U(iii) metallocene complexes.^75^ The O1–U1–C36 bond angle is 96.97(11)° and 3 has a 172.7(2)° U1–O1–C(ipso) angle. The U–O(aryloxide) bond length of 2.166(2) Å is shorter than in other U(iii) metallocene aryloxide complexes. For example, the U–O(aryloxide) bond distance in 1 is 2.229(3) Å and 2.201(6) Å in 2, but nearly identical to the ∼2.157 Å for the average U–O distances in U(O-2,4,6-^t^Bu_3_C_6_H_2_)_3_.^61^ Commensurate with the amount of backbonding observed from the IR stretching frequencies, the U–C(CO) bond distance in 3 is 2.394(5) Å which is slightly longer than the 2.383(6) Å in 3b and much shorter than the 2.485(9) Å in 3a. Interestingly, the C–O bond distance is 1.363(4) Å which is substantially longer than that of free CO (1.128 Å),^70^ and longer than those observed in 3a and 3b of 1.13(1) and 1.142(7) Å, respectively. Finally, the U–C–O bond angle is 177.8(4)° compared to 180° and 175.2(6)° in 3a and 3b, respectively.

The electronic structure of 3 was probed using DFT calculations (B3PW91). Different spin states were considered (doublet, quartet, and sextet) and the geometry was optimized in all cases. The quartet, in line with a U(iii) centre, is found to be the ground state with the doublet 13.8 kcal mol^−1^ higher in energy and finally the sextet 55.5 kcal mol^−1^ above the ground state, Table S3.† This is consistent with the UV-vis-nIR spectrum (Fig. S3†) which shows similar features to 1.^76^ The optimized geometry compares well with the experimental one with a maximum deviation of 0.03 Å on the U–C_CO_ distance. The CO bond appears to be slightly elongated 0.03 Å with respect to free CO. This slight elongation accounts for the low CO stretching frequency (1899 cm^−1^). The bonding was thus analysed using the Natural Bonding Orbital (NBO) analysis. A U–C bond polarized toward C (75%) is observed and this is further corroborated by the low Wiberg Bond Index (WBI) of 0.48 indicating a reduced covalency in the bond (for the sake of comparison, the U–O WBI is 0.40 and the U-Cp ones are 0.11). This bond is a σ bond that involves the overlap of a hybrid sp orbital on C and a hybrid *spdf* (15–11–55–19). The C–O bond is found to be a double bond polarized toward O (70% for the σ and 76% for the π) with a WBI of 1.05, in line with the polarization of the bond. To probe the mechanism for back donation, a large core structure of 3 was optimized. Large core structures are obtained with f-in-core Relativistic Core Potentials (RCPs) where the f electron configuration is fixed and adapted to a given oxidation state, in this case 5f^3^ for U(iii). The large core calculations do not allow any back donation from the metal since the f electrons are not explicitly treated. The large core structure gave a stretching frequency of 2098 cm^−1^, only 12 cm^−1^ lower than that of free CO. In contrast, for the small core structure, in which the f electrons are treated explicitly, a stretching frequency of 1899 cm^−1^ was found. Therefore, backbonding observed in 3 is primarily due to the SOMO-2 orbital (20% 5f, 55% 6d), Fig. S18,† with a minor contribution of the (C_5_Me_5_)^1−^ to CO interaction, Fig. S19.†

Next, we examined the reactivity of CO with a less sterically crowded aryloxide, *i.e.*, a mesityl group. Addition of 1 atm of CO to 2 in pentane at ambient temperature led to a colour change from dark green to black. A red powder was isolated in excellent (89%) yield. The solid-state structure was determined by X-ray diffraction analysis to reveal the bridging ethynediolate moiety, [{(C_5_Me_5_)_2_(MesO)U}_2_(*μ*_2_-OC≡CO)], 4. The fact that the aryloxide with larger steric properties prevented C–C bond formation indicates that the probable zig-zag intermediate^54^ cannot form due to the steric properties of the BHT ligand in 1, so only coordination occurs to form 3. In comparison to Cloke's system, [{C_8_H_6_(Si^*i*^Pr_3_)_2_}(C_5_Me_5_)U], where CO coordination was observed, quickly followed by ethynediolate formation, indicates that its steric properties are probably between those of 1 and 2.2
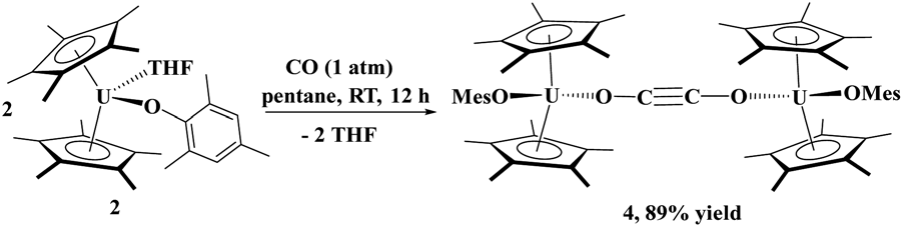


**Fig. 2:**
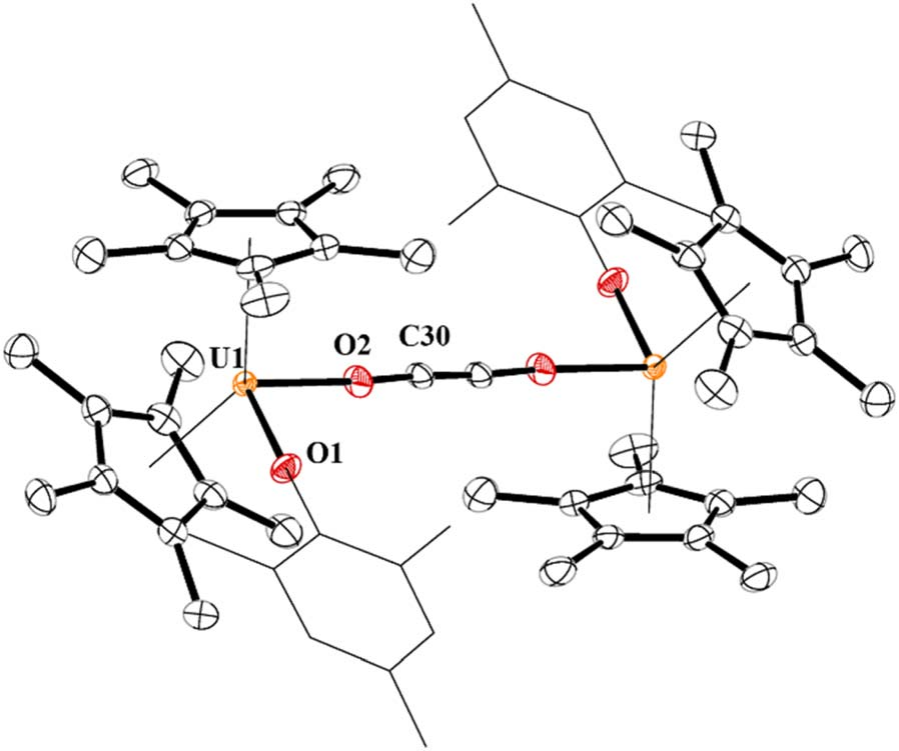
Thermal ellipsoid plot of 4 shown at the 50% probability level. The hydrogen atoms have been omitted and the mesityl group is shown in wireframe for clarity.

In 4, each uranium adopts a pseudo-tetrahedral geometry with an inversion center through the molecule making one unique set of distances and angles, Fig. 2. The U–O(OMes) and U–O(OC) bond distances of 2.122(2) and 2.129(2) Å, respectively, as well as the U–O–C(ipso) angle of 166.18(2)° are typical of other U(iv)–O aryloxide complexes. The C–C bond length of 1.21(1) Å in 4 is longer than the 1.177(12), 1.183(7), and 1.187(8) Å in the other three U(iv) ethynediolate complexes, but identical to the 1.226(10) Å in [{(1,2,4-^t^Bu_3_C_5_H_2_)_2_Tm}_2_(*μ*-OCCO)].^34^ Concomitant with the lengthening of the C–C bond is the slight decrease in the C–O bond distance of 1.280(7) Å which can also be compared to 1.296(10), 1.301(4), and 1.302(5) Å in the other ethynediolates. Additionally, the U–O2–C30 bond angle in 4 is 172.4(3)° and a C30′-C30-O2 angle of 176.3(7)°.

A weak broad absorption in the IR spectrum at 2010 cm^−1^ is observed for 4, consistent with an alkyne which is asymmetric in the solid-state. The ^1^H NMR spectrum showed resonances ranging from −31 to +2.54 ppm, characteristic of a paramagnetic complex. While the solid-state structure had only one set of unique bonds and angles, the ^1^H NMR spectrum revealed an asymmetric mesityl group with resonances for the *ortho*-methyl groups at −17.76 and −30.98 ppm, the *para*-methyl at 0.05 ppm, and the *meta*-hydrogens at 1.48 and 2.54 ppm. The reaction was also conducted with ^13^CO which produced a resonance at 310 ppm, very similar to the 314.2 ppm observed in [{{C_8_H_6_(Si^i^Pr_3_)_2_}(C_5_Me_5_)U}_2_(*μ*-OCCO)].^54^

**Fig. 3:**
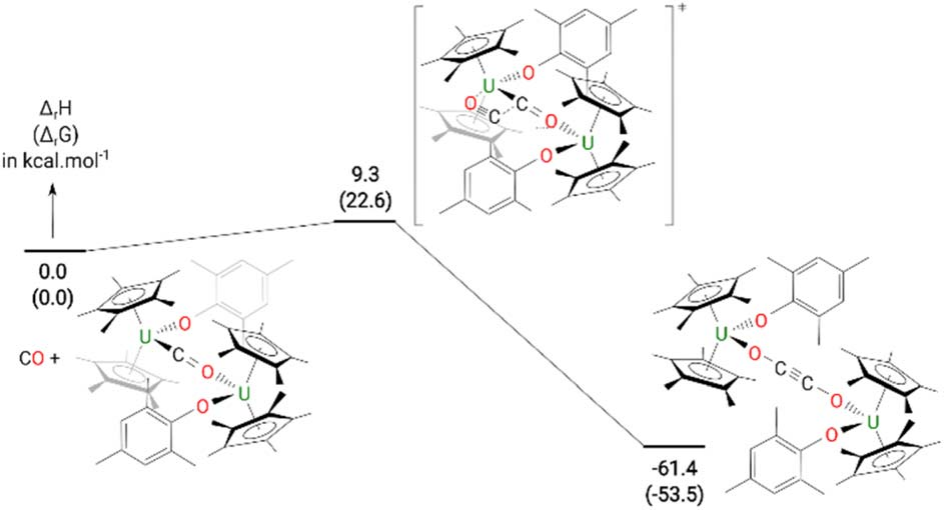
Computed enthalpy (Gibbs free energies are given in brackets) for the formation of 4 at room temperature. The energies are given in kcal mol^−1^.

The formation of 4 was investigated computationally at the DFT level. The reaction (Fig. 3) begins by the formation of the so-called key intermediate where a doubly-reduced CO molecule is sandwiched in between two uranium centres. This diuranium complex is slightly destabilized by 9.4 kcal mol^−1^ in enthalpy (7.7 kcal mol^−1^ in Gibbs Free energy) with respect to the separated reactant. Different spin states were considered for this intermediate to check the degree of reduction of the CO molecule. The ground state is a quintet spin state, in line with the presence of two U(iv) and therefore a doubly-reduced CO. This reduction of the CO bond is further highlighted by the CO bond length (1.26 Å) which is elongated by 0.11 Å with respect to 3. The activated CO can then react with another CO molecule, *via* a low-lying transition state (TS) with an associated barrier of 9.3 kcal mol^−1^. This C–C coupling TS is better described as a CO insertion reaction onto the U–C bond with a classical 4-member ring TS, as opposed to a zig-zag like moiety. At the TS, the C–C bond is not yet formed (1.88 Å) while the C–O bond of the insertion CO is elongated to 1.22 Å with a U–O distance of 2.34 Å. Following the intrinsic reaction coordinate, it yields complex 4 whose formation is exothermic by 61.4 kcal mol^−1^. While the solid-state structure of 4 showed the mesityl groups on opposite sides to each other, calculations indicate that the isomer with the mesityl groups on the same side is only 0.4 kcal mol^−1^ higher in energy in the gas phase, Fig. S21.† However, only the energy states were calculated, and a variable temperature NMR experiment did not show the other isomer.

**Scheme 1:**
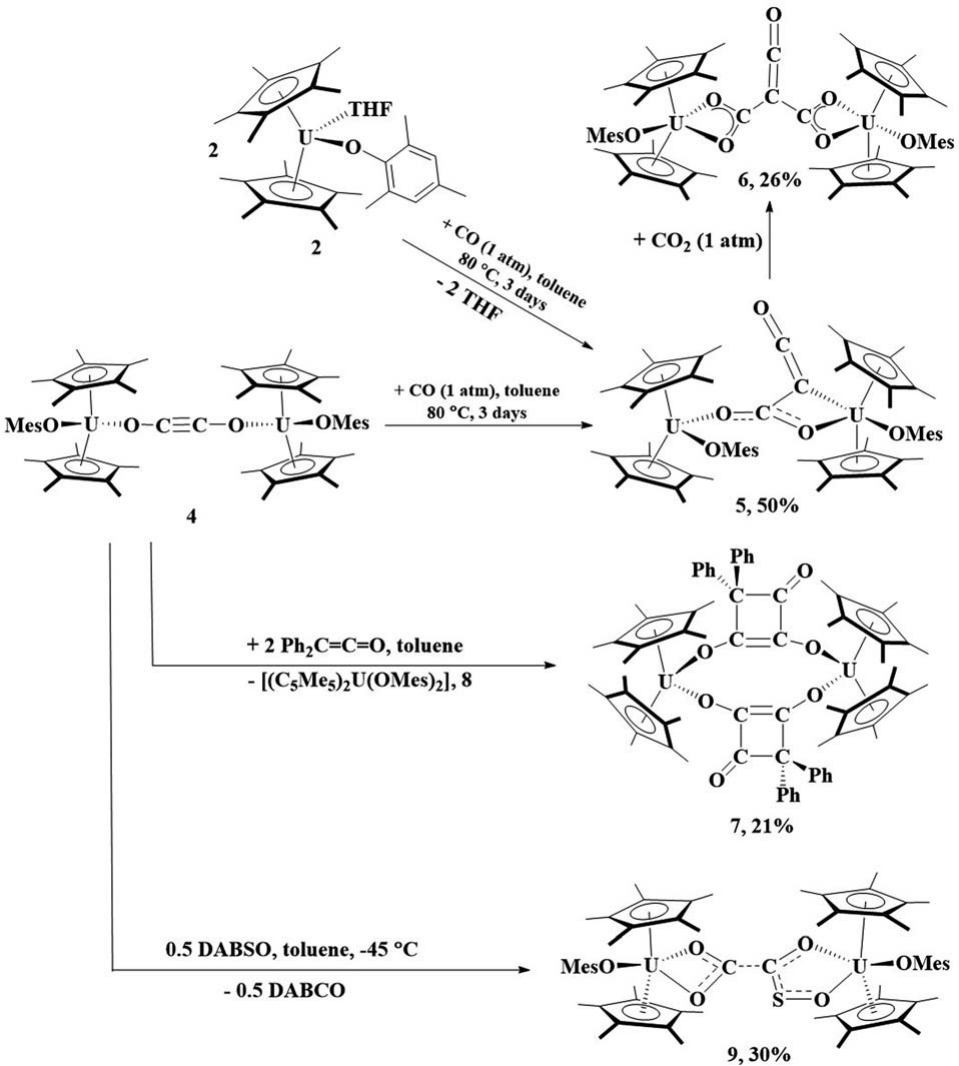
Reactivity of 4 with CO, Ph_2_CCO, and DABSO as well as 5 with CO_2_. Percentages reflect crystalline yields obtained.

**Fig. 4:**
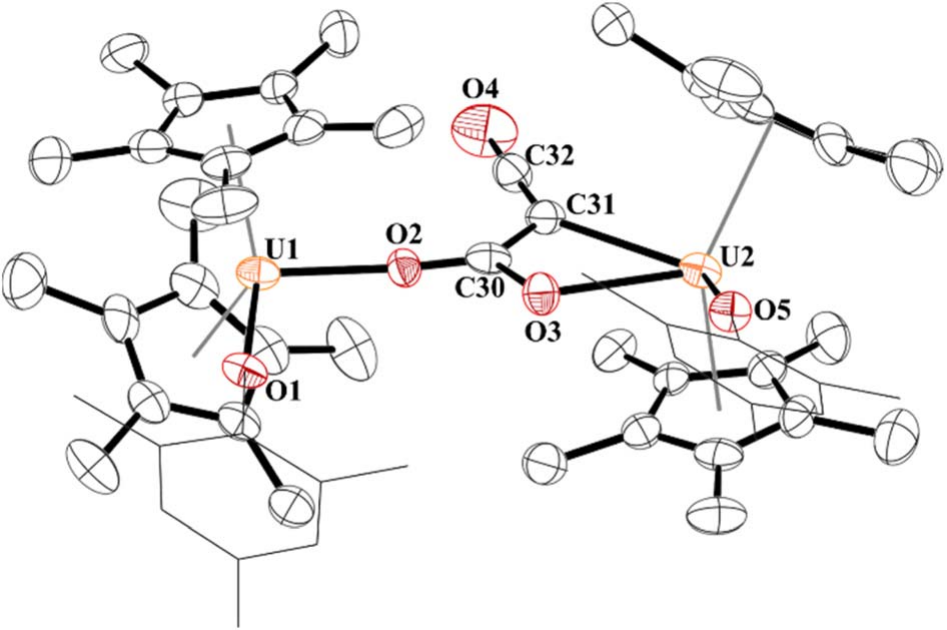
Thermal ellipsoid plot of 5 shown at the 50% probability level. The hydrogen atoms have been omitted and the mesityl group is shown in wireframe for clarity.

The reaction of 2 with CO can be heated to 80 °C for 3 days, resulting in the formation of 5, Scheme 1. Complex 5 can be obtained from isolation of 4, followed by treatment with 1 atm CO. Orange crystals suitable for X-ray diffraction analysis were grown from a saturated pentane solution at −25 °C. The solid-state structure of 5 revealed a ketenecarboxylate bridging two uranium(iv) metal centers in which one oxygen of the carboxylate is bound *κ*^1^ to one uranium (U1) with a U–O2 bond distance of 2.137(6) Å, while the other oxygen (O3) is coordinated to U2 with a distance of 2.461(6) Å, Fig. 4. The U2–C31 bond length of 2.639(9) Å is long for a U–C bond. Therefore, the geometry about U1 is pseudo-tetrahedral while U2 is a distorted trigonal bipyramidal. The O1–U1–O2 bond angle is 102.2(2)°, the C31–U2–O3 angle is 51.8(2)°, and the C31–U2–O5 and O3–U2–O5 angles are 131.8(2)° and 81.67(19)°, respectively. Consistent with a ketene moiety, the C31–C32 bond distance is 1.295(13) Å, consistent with the C–C double bond, with a short C32–O4 length of 1.193(11) Å, assigned as a carbonyl. A longer C31–C30 distance of 1.442(11) Å, a C–C single bond, and C30–O2 and C30–O3 lengths of 1.233(8) and 1.297(8) Å, respectively, are consistent with a delocalized carboxylate. The U–O(aryloxide) bond lengths of 2.075(4) and 2.114(4) Å are expected for U(iv) aryloxide complexes.

The ^1^H NMR spectrum of 5 features two resonances for each proton environment indicating an asymmetric ligand system. For example, two (C_5_Me_5_)^1−^ resonances are observed at −0.37 and −0.13 ppm. In addition, four *ortho*-methyl resonances are located at −35.31, −25.52, −21.13, and −12.40 ppm. A ketene stretch at 2065 cm^−1^, characteristic for a ketene moiety, is seen in the IR spectrum.

**Fig. 5:**
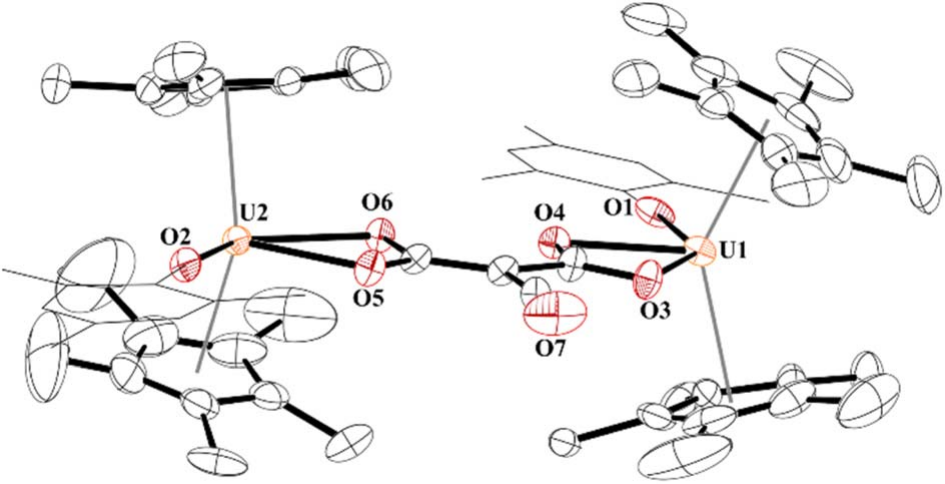
Thermal ellipsoid plot of 6 shown at the 50% probability level. The hydrogen atoms have been omitted and the mesityl group is shown in wireframe for clarity.

Since complex 5 contained a uranium–carbon bond which is well known to undergo insertion chemistry,^77–79^ we reacted 5 with CO_2_. The result is indeed the product of CO_2_ insertion into the uranium–carbon bond in 5 forming a ketenedicarboxylate, [{(C_5_Me_5_)_2_(OMes)U}_2_(*μ*_2_:*κ*2:*κ*2-(O,O)–C_4_O_5_)], 6, Fig. 5. Again, this is the same moiety obtained by Nocton and co-workers with thulium. Although the crystal structure refinement of most light atoms in 6 is severely affected by the non-ideal nature of the crystal, there is evidence that the ketenedicarboxylate itself is not severely impacted by error. In particular, the unusually wide OOC-C-COO bond angle of 131.7(6)^o^ observed in the Tm complex is reproduced in 6 (130.4(2)^o^). The U⋯U distance (7.824(1) Å) is also very comparable to the reported Tm⋯Tm distance (7.6583(9) Å) and slightly longer than U⋯U distances in alpha-dicarboxylates (7.167 to 7.678 Å), which were previously only reported for U(vi).^41,80–82^ Complex 6 has U–O(aryloxide) bond distances of 2.109(15) and 2.099(14) Å with each aryloxide ligand positioned on opposite sides of the molecule from each other. Both sets of carboxylate groups have identical C–O bonds: C59–O3, 1.28(3) Å; C59–O4, 1.27(2) Å; C61–O5, 1.26(2) Å; C61–O6, 1.28(2) Å. These are significantly longer than the C–O double bond in the ketene (C62–O7) of 1.07(3) Å.

The ^1^H NMR spectrum of 6 shows the (C_5_Me_5_)^1−^ resonance at −0.89 ppm and the *ortho*-methyl groups at 17.44 and 44.24 ppm. However, the *para*-methyl groups could not be located. We did not observe the two asymmetric ketene stretching vibrations in the IR spectrum. This might be due to that the C–C bond distance of the ketene moiety in 6 is 1.40(3) Å compared to 1.343(10) Å in the analogous thulium complex. We do observe the absorption band at 1474 cm^−1^ that we assign for the carboxylate groups.

We next investigated electrophilic substrates capable of [2 + 2] cycloaddition such as diphenylketene and SO_2_. Reaction of 4 with Ph_2_C=C=O results in [{(C_5_Me_5_)_2_U}_2_(OC(CPh_2_)C(=O)CO)], 7, a 1,2-dioxy-4,4-diphenylcyclobut-2-en-1-one bridge between two U(iv) centres with concomitant formation of the ligand distribution product, [(C_5_Me_5_)_2_U(OMes)_2_], 8, Scheme 1. This is a rare example of a cycloaddition reaction from CO homologation, and first with an ethynediolate, and, like complex 5, increases the carbon chain to a C4 product. Complex 7 is also an unusual example of [2 + 2] cycloaddition with a metal complex that does not result in a metallocycle. Complex 8 is readily obtained from the reaction of [(C_5_Me_5_)_2_UCl_2_] with two equivalents of KOMes.

**Fig. 6:**
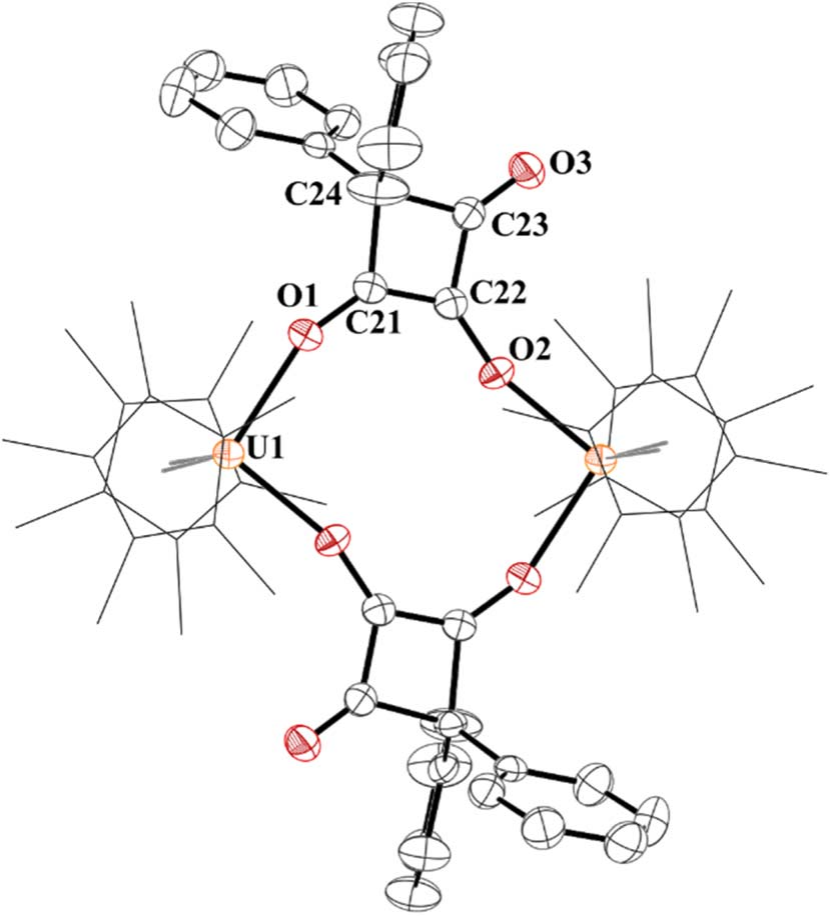
Thermal ellipsoid plot of 7 shown at the 50% probability level. The hydrogen atoms have been omitted and (C_5_Me_5_)^1-^ ligands are shown in wireframe for clarity.

The structure of 7 has a pseudo-tetrahedral geometry about each uranium centre with an inversion making only one set of unique bond distances and angles, Fig. 6. The C21–C22 distance of 1.359(5) Å can be compared to the C21–C24, C22–C23, and C23–C24 bond lengths which are 1.541(5) Å, 1.436(5), and 1.568(5) Å, respectively. Therefore, C21–C22 can be assigned as a C–C double bond, while the others are consistent with C–C single bonds, although the 1.436(5) Å is in between a double and single bond. The C–O bond distances, C21–O1 and C22–O2 are 1.295(4) and 1.330(4) Å, respectively, assignable as C–O single bonds while C23–O3 is a carbonyl with a C–O bond distance of 1.207(4) Å.

The ^1^H NMR spectrum was taken in CD_2_Cl_2_ due to the poor solubility in C_6_D_6_ of 7 and showed a (C_5_Me_5_)^1−^ resonance at 5.47 ppm. The phenyl protons were paramagnetically shifted from −11.41 to 5.06 ppm. The ^1^H NMR spectrum of 8 has a (C_5_Me_5_)^1−^ resonance at 3.59 ppm, *ortho*-methyl resonances at −8.79 and −3.16 ppm, and *para*-methyl at 2.47 ppm. A weak absorption at 1752 cm^−1^ is attributed to the carbonyl group. The structure of 8 was also determined and shows similar metrical parameters to other U(iv) metallocene bis(aryloxide) complexes.^83^

Finally, while not extending the carbon chain, another substrate capable of [2 + 2] addition, *i.e.*, electrophile SO_2_, was obtained using half an equivalent of 1,4-diazabicyclo[2,2,2]octane bis(sulfur dioxide) adduct, DABSO, Scheme 1. Very few molecular examples of SO_2_ chemistry are reported with f elements.^84–89^ To our surprise, the product, 10, involves the cleavage of a S–O bond to form a thiocarbonyl,^90,91^ a rare accomplishment and testament to the highly nucleophilic nature of the ethynediolate, to yield an unprecedented [O_2_CC(O)(SO)]^2−^ ligand bridging between two [(C_5_Me_5_)_2_(MesO)U]^1+^ motifs. Further, thiocarbonyl (SO) is rarely isolated in the reactivity of SO_2_ but often invoked in its reduction.

**Fig. 7:**
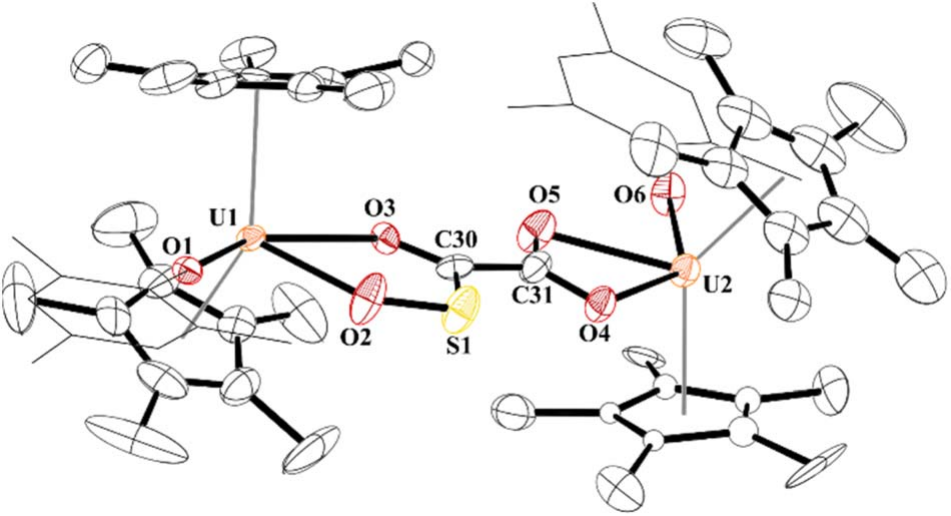
Thermal ellipsoid plot of 9 shown at the 50% probability level. The hydrogen atoms have been omitted and the mesityl group is shown in wireframe for clarity.

While the quality of the data is not ideal and displays large error limits, we can extract enough metrics to determine the correct connectivity of the atoms, Fig. 7. The carboxylate at one uranium(iv) metal centre in 9 has U2–O distances of 2.493(8) and 2.444(8) Å, typical of other U(iv) carboxylate bond distances.^92^ However, the U1–O bond distances are 2.486(8) and 2.338(8) Å. The latter, while shorter than the other three U–O bonds, is the same as the 2.339(2) Å in [(C_5_Me_5_)(^Mes^PDI^Me^)U{O=P(NMes_2_)_3_}], ^Mes^PDI^Me^ = 2,6-{(Mes)N=CMe}_2_C_5_H_3_N, with a strong donor ligand (HMPA).^93^ For example, The C30–C31 bond distance that was formally a C–C triple bond at 1.223(7) Å in 4 is now 1.491(17) Å in 9 and assigned as a C–C single bond. The C–O bond distances are all the same: O4–C31, 1.250(14) Å; O5–C31, 1.262(14) Å; O3–C30, 1.267(14) Å, indicating a single and double bond character. The C–S length is 1.683(13) Å which is similar to the 1.67(2) and 1.76(2) Å in [{(C_5_Me_5_)_2_(MesO)U}_2_(*μ*-CS_2_)]. Furthermore, the S–O bond of 1.551(9) Å is in between the 1.634(1) Å and 1.476(1) Å observed in ^*t*^Bu_2_P(=O)–S(=O)–O–B(N^i^Pr_2_)_2_,^94^ and nearly identical to that observed in sulfinates formed by SO_2_ insertion into Zn–C bonds.^95,96^ From these metric parameters, we conclude that the two chelates to each uranium are both fully delocalized, like the coordination of an oxalate dianion.^97^

**Fig. 8:**
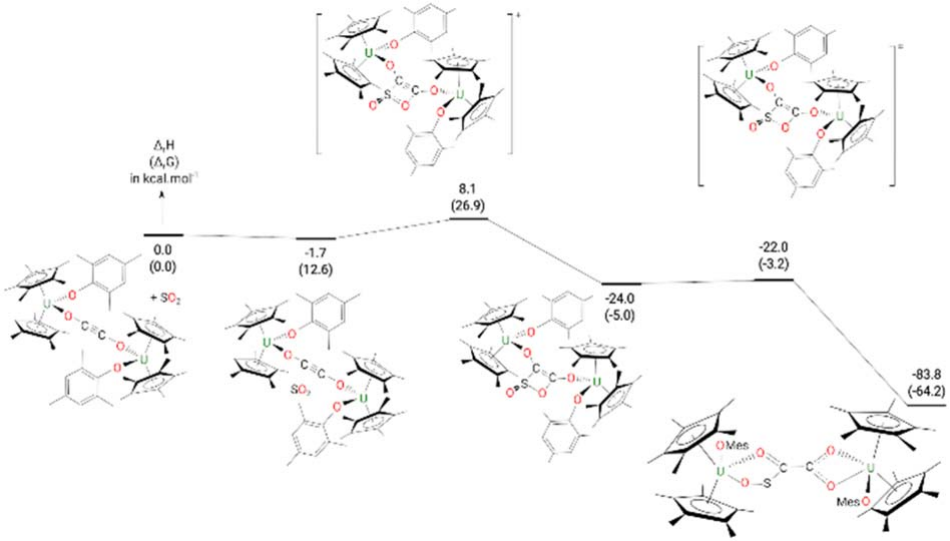
Computed enthalpy (Gibbs free energies are given in brackets) for the formation of 9 at room temperature. The energies are given in kcal mol^−1^.

The formation of complex 9 from complex 4 (Fig. 8) begins by the formation of a van der Waals adduct of SO_2_ which is marginally stabilized by 1.7 kcal mol^−1^. From this adduct, the system undergoes a [2 + 2] cycloaddition with an associated low barrier of 9.8 kcal mol^−1^. The [2 + 2] cycloaddition nature of the reaction is highlighted by the orientation of the SO_2_ molecule at the TS, which lies in a plane parallel to the equatorial plane of the ethynediolate in 4. In such an orientation, the reaction implies the π system of the two molecules (4 and SO_2_). At the TS, the C–S bond is almost formed (1.87 Å) while the C–O one remains long (2.24 Å). Following the intrinsic reaction coordinate, it yields the formation of a cycloaddition (4-membered ring) intermediate which, although stable by 24.0 kcal mol^−1^, readily evolves by breaking the S–O bond involved in the 4-member ring. The associated barrier is 2.0 kcal mol^−1^, indicating a very facile reaction. This low-lying TS allows the formation of the very stable complex 9 (−83.8 kcal mol^−1^).

## Conclusions

In summary, we have examined the reactivity of two heteroleptic metallocene aryloxide uranium(iii) complexes with CO. In the case of the larger aryloxide, only a coordination complex was observed and structurally characterized. However, no further reactivity was observed indicating that the steric bulk of the aryloxide did not allow for homologation to occur. With the sterically less bulky mesityl group, homologation to form an ethynediolate occurred. The reactivity of the ethynediolate complex was probed with additional CO, Ph_2_C=C=O, and SO_2_ (DABSO). While there was a precedent for the CO reactivity, the [2 + 2] cycloaddition obtained with Ph_2_CCO and the S–O bond cleavage seen with SO_2_ afforded novel and unusual reactivity. This establishes the potential for uranium to be involved in carbon chain growth chemistry directly from CO, as well as the ability for the ethynediolate, formed by CO homologation, to subsequently undergo unique transformations.

## Conflicts of interest

There are no conflicts to declare.

## Data availability

Experimental procedures, spectroscopic data, information on the theoretical calculations and crystallographic details can be found in the ESI.†

## Author contributions

R. J. Ward performed the synthetic experimental work; I. Del Rosal and L. Maron performed and interpreted the computational studies; S. P. Kelley recorded and interpreted the X-ray diffraction analysis; J. R. Walensky conceptualized the research, acquired funding, and supervised the work; All authors revised and edited the manuscript. All authors have read and agreed to the published version of the manuscript.

## Supplementary Material

SC-014-D2SC06375A-s001

SC-014-D2SC06375A-s002

SC-014-D2SC06375A-s003

## References

[cit1] Maitlis P. M. (2004). J. Organomet. Chem..

[cit2] West N. M., Miller A. J. M., Labinger J. A., Bercaw J. E. (2011). Coord. Chem. Rev..

[cit3] Labinger J. A. (2017). J. Organomet. Chem..

[cit4] Batuecas M., Kong R. Y., White A. J. P., Crimmin M. R. (2022). Angew. Chem., Int. Ed..

[cit5] Heilmann A., Roy M. M. D., Crumpton A. E., Griffin L. P., Hicks J., Goicoechea J. M., Aldridge S. (2022). J. Am. Chem. Soc..

[cit6] Kong R. Y., Crimmin M. R. (2020). Dalton Trans..

[cit7] Wayland B., Fu X. (2006). Science.

[cit8] Hasegawa S., Ishida Y., Kawaguchi H. (2021). Chem. Commun..

[cit9] Maron L., Eisenstein O., Andersen R. A. (2009). Organometallics.

[cit10] Arnold P. L. (2011). Chem. Commun..

[cit11] Arnold P. L., Turner Z. R. (2017). Nat. Rev. Chem..

[cit12] Barluzzi L., Giblin S. R., Mansikkamäki A., Layfield R. A. (2022). J. Am. Chem. Soc..

[cit13] Gmelin L. (1825). Ann. Phys..

[cit14] LIEBIG J. (1834). Ann. Pharm..

[cit15] Büchner W. (1963). Helv. Chim. Acta.

[cit16] Coluccia S., Garrone E., Guglielminotti E., Zecchina A. (1981). Trans. Faraday Soc..

[cit17] Lednor P. W., Versloot P. C. (1983). J. Chem. Soc., Chem. Commun..

[cit18] Lalrempuia R., Kefalidis C. E., Bonyhady S. J., Schwarze B., Maron L., Stasch A., Jones C. (2015). J. Am. Chem. Soc..

[cit19] Anker M. D., Hill M. S., Lowe J. P., Mahon M. F. (2015). Angew. Chem., Int. Ed..

[cit20] Yuvaraj K., Douair I., Paparo A., Maron L., Jones C. (2019). J. Am. Chem. Soc..

[cit21] Yuvaraj K., Douair I., Jones D. D. L., Maron L., Jones C. (2020). Chem. Sci..

[cit22] Paparo A., Yuvaraj K., Matthews A. J. R., Douair I., Maron L., Jones C. (2021). Angew. Chem., Int. Ed..

[cit23] Braunschweig H., Dellermann T., Dewhurst R. D., Ewing W. C., Hammond K., Jimenez-Halla J. O. C., Kramer T., Krummenacher I., Mies J., Phukan A. K., Vargas A. (2013). Nat. Chem..

[cit24] Wang X., Zhu Z., Peng Y., Lei H., Fettinger J. C., Power P. P. (2009). J. Am. Chem. Soc..

[cit25] Brown Z. D., Power P. P. (2013). Inorg. Chem..

[cit26] Majumdar M., Omlor I., Yildiz C. B., Azizoglu A., Huch V., Scheschkewitz D. (2015). Angew. Chem., Int. Ed..

[cit27] Kong R. Y., Crimmin M. R. (2018). J. Am. Chem. Soc..

[cit28] Protchenko A. V., Vasko P., Do D. C. H., Hicks J., Fuentes M. Á., Jones C., Aldridge S. (2019). Angew. Chem., Int. Ed..

[cit29] Xiong Y., Yao S., Szilvási T., Ruzicka A., Driess M. (2020). Chem. Commun..

[cit30] Evans W. J., Grate J. W., Hughes L. A., Zhang H., Atwood J. L. (1985). J. Am. Chem. Soc..

[cit31] Evans W. J., Lee D. S., Ziller J. W., Kaltsoyannis N. (2006). J. Am. Chem. Soc..

[cit32] Fang M., Farnaby J. H., Ziller J. W., Bates J. E., Furche F., Evans W. J. (2012). J. Am. Chem. Soc..

[cit33] Ryan A. J., Ziller J. W., Evans W. J. (2020). Chem. Sci..

[cit34] Simler T., McCabe K. N., Maron L., Nocton G. (2022). Chem. Sci..

[cit35] Moloy K. G., Marks T. J., Day V. W. (1983). J. Am. Chem. Soc..

[cit36] Sonnenberger D. C., Mintz E. A., Marks T. J. (1984). J. Am. Chem. Soc..

[cit37] Fagan P. J., Moloy K. G., Marks T. J. (1981). J. Am. Chem. Soc..

[cit38] Manriquez J. M., Fagan P. J., Marks T. J., Day C. S., Day V. W. (1978). J. Am. Chem. Soc..

[cit39] Weydert M., Brennan J. G., Andersen R. A., Bergman R. G. (1995). Organometallics.

[cit40] Fagan P. J., Manriquez J. M., Marks T. J., Day V. W., Vollmer S. H., Day C. S. (1980). J. Am. Chem. Soc..

[cit41] Katahira D. A., Moloy K. G., Marks T. J. (1982). Organometallics.

[cit42] Moloy K. G., Fagan P. J., Manriquez J. M., Marks T. J. (1986). J. Am. Chem. Soc..

[cit43] Dormond A., Aaliti A., Elbouadili A., Moise C. (1987). J. Organomet. Chem..

[cit44] Arnold P. L., Turner Z. R., Germeroth A. I., Casely I. J., Nichol G. S., Bellabarba R., Tooze R. P. (2013). Dalton Trans..

[cit45] Bénaud O., Berthet J.-C., Thuéry P., Ephritikhine M. (2010). Inorg. Chem..

[cit46] Evans W. J., Siladke N. A., Ziller J. W. (2010). Chem.–Eur. J..

[cit47] Edwards P. G., Hursthouse M. B., Malik K. M. A., Parry J. S. (1994). J. Chem. Soc., Chem. Commun..

[cit48] Edwards P. G., Parry J. S., Read P. W. (1995). Organometallics.

[cit49] Fagan P. J., Manriquez J. M., Vollmer S. H., Day C. S., Day V. W., Marks T. J. (1981). J. Am. Chem. Soc..

[cit50] Vilanova S. P., del Rosal I., Tarlton M. L., Maron L., Walensky J. R. (2018). Angew. Chem., Int. Ed..

[cit51] Tarlton M. L., Del Rosal I., Vilanova S. P., Kelley S. P., Maron L., Walensky J. R. (2020). Organometallics.

[cit52] Summerscales O. T., Cloke F. G. N., Hitchcock P. B., Green J. C., Hazari N. (2006). Science.

[cit53] Summerscales O. T., Cloke F. G. N., Hitchcock P. B., Green J. C., Hazari N. (2006). J. Am. Chem. Soc..

[cit54] Frey A. S., Cloke F. G. N., Hitchcock P. B., Day I. J., Green J. C., Aitken G. (2008). J. Am. Chem. Soc..

[cit55] Aitken G., Hazari N., Frey A. S. P., Cloke F. G. N., Summerscales O., Green J. C. (2011). Dalton Trans..

[cit56] McKay D., Frey A. S. P., Green J. C., Cloke F. G. N., Maron L. (2012). Chem. Commun..

[cit57] Tsoureas N., Summerscales O. T., Cloke F. G. N., Roe S. M. (2013). Organometallics.

[cit58] Kahan R. J., Farnaby J. H., Tsoureas N., Cloke F. G. N., Hitchcock P. B., Coles M. P., Roe S. M., Wilson C. (2018). J. Organomet. Chem..

[cit59] Arnold P. L., Turner Z. R., Bellabarba R. M., Tooze R. P. (2011). Chem. Sci..

[cit60] Van der Sluys W. G., Burns C. J., Huffman J. C., Sattelberger A. P. (1988). J. Am. Chem. Soc..

[cit61] Mansell S. M., Kaltsoyannis N., Arnold P. L. (2011). J. Am. Chem. Soc..

[cit62] Gardner B. M., Stewart J. C., Davis A. L., McMaster J., Lewis W., Blake A. J., Liddle S. T. (2012). Proc. Natl. Acad. Sci. U.S.A..

[cit63] Castro-Rodriguez I., Meyer K. (2005). J. Am. Chem. Soc..

[cit64] Arnold P. L. and Mansell S. M., CSD Communication, CCDC 1487672, 2016, 10.5517/ccdc.csd.cc11y1f0

[cit65] Cleaves P. A., King D. M., Kefalidis C. E., Maron L., Tuna F., McInnes E. J. L., McMaster J., Lewis W., Blake A. J., Liddle S. T. (2014). Angew. Chem., Int. Ed..

[cit66] Falcone M., Chatelain L., Scopelliti R., Živković I., Mazzanti M. (2017). Nature.

[cit67] Palumbo C. T., Barluzzi L., Scopelliti R., Zivkovic I., Fabrizio A., Corminboeuf C., Mazzanti M. (2019). Chem. Sci..

[cit68] Wedal J. C., Ziller J. W., Furche F., Evans W. J. (2022). Inorg. Chem..

[cit69] Ward R. J., Pividori D., Carpentier A., Tarlton M. L., Kelley S. P., Maron L., Meyer K., Walensky J. R. (2021). Organometallics.

[cit70] Cotton F. A. and Wilkinson G., Advanced inorganic chemistry: A comprehensive text, John Wiley & Sons, Nashville, TN, 5 edn, 1988

[cit71] Brennan J. G., Andersen R. A., Robbins J. L. (1986). J. Am. Chem. Soc..

[cit72] Tarlton M. L., Yu X., Ward R. J., Kelley S. P., Autschbach J., Walensky J. R. (2021). Chem.–Eur. J..

[cit73] Evans W. J., Kozimor S. A., Nyce G. W., Ziller J. W. (2003). J. Am. Chem. Soc..

[cit74] Parry J., Carmona E., Coles S., Hursthouse M. (1995). J. Am. Chem. Soc..

[cit75] Wedal J. C., Windorff C. J., Huh D. N., Ryan A. J., Ziller J. W., Evans W. J. (2021). J. Coord. Chem..

[cit76] Ward R. J., Kelley S. P., Lukens W. W., Walensky J. R. (2022). Organometallics.

[cit77] Matson E. M., Forrest W. P., Fanwick P. E., Bart S. C. (2011). J. Am. Chem. Soc..

[cit78] Matson E. M., Fanwick P. E., Bart S. C. (2011). Organometallics.

[cit79] Evans W. J., Walensky J. R., Ziller J. W. (2010). Organometallics.

[cit80] Zhang Y., Livens F. R., Collison D., Helliwell M., Heatley F., Powell A. K., Wocadlo S., Eccles H. (2002). Polyhedron.

[cit81] Zhang Y., Collison D., Livens F. R., Helliwell M., Heatley F., Powell A. K., Wocadlo S., Eccles H. (2002). Polyhedron.

[cit82] Medvedkov Y. A., Grigorev M. S., Serezhkina L. B., Pushkin D. V., Serezhkin V. N. (2018). Russ. J. Inorg..

[cit83] Evans W. J., Miller K. A., DiPasquale A. G., Rheingold A. L., Stewart T. J., Bau R. (2008). Angew. Chem., Int. Ed..

[cit84] Benndorf P., Schmitt S., Köppe R., Oña-Burgos P., Scheurer A., Meyer K., Roesky P. W. (2012). Angew. Chem., Int. Ed..

[cit85] Klementyeva S. V., Gamer M. T., Schmidt A.-C., Meyer K., Konchenko S. N., Roesky P. W. (2014). chem. eur. j..

[cit86] Schmidt A.-C., Heinemann F. W., Kefalidis C. E., Maron L., Roesky P. W., Meyer K. (2014). Chem.–Eur. J..

[cit87] Klementyeva S. V., Arleth N., Meyer K., Konchenko S. N., Roesky P. W. (2015). New J. Chem..

[cit88] Schoo C., Klementyeva S. V., Gamer M. T., Konchenko S. N., Roesky P. W. (2016). Chem. Commun..

[cit89] Louyriac E., Roesky P. W., Maron L. (2017). Dalton Trans..

[cit90] Buß F., Rotering P., Mück-Lichtenfeld C., Dielmann F. (2018). Dalton Trans..

[cit91] Logdi R., Bag A., Tiwari A. K. (2022). J. Phys. Chem. A.

[cit92] Thierry L., Ionut M., Natacha H., Christophe V. (2014). Coord. Chem. Rev..

[cit93] Kiernicki J. J., Cladis D. P., Fanwick P. E., Zeller M., Bart S. C. (2015). J. Am. Chem. Soc..

[cit94] Szynkiewicz N., Chojnacki J., Grubba R. (2020). Inorg. Chem..

[cit95] Kelly R. P., Kazeminejad N., Lamsfus C. A., Maron L., Roesky P. W. (2016). Chem. Commun..

[cit96] Tulewicz A., Szejko V., Justyniak I., Wolska-Pietkiewicz M., Lewiński J. (2022). Dalton Trans..

[cit97] Inman C. J., Frey A. S. P., Kilpatrick A. F. R., Cloke F. G. N., Roe S. M. (2017). Organometallics.

