# Correction: Isolation of C1 through C4 derivatives from CO using heteroleptic uranium(iii) metallocene aryloxide complexes

**DOI:** 10.1039/d3sc90046k

**Published:** 2023-03-09

**Authors:** Robert J. Ward, Iker del Rosal, Steven P. Kelley, Laurent Maron, Justin R. Walensky

**Affiliations:** a Department of Chemistry, University of Missouri Columbia MO 65211 USA walenskyj@missouri.edu; b Universite de Toulouse, CNRS, INSA, UMR, UMR 5215 LPCNO 135 Avenue de Ranguiel 31077 Toulouse France

## Abstract

Correction for ‘Isolation of C1 through C4 derivatives from CO using heteroleptic uranium(iii) metallocene aryloxide complexes’ by Robert J. Ward *et al.*, *Chem. Sci.*, 2023, **14**, 2024–2032, https://doi.org/10.1039/D2SC06375A.

The authors regret that the C–O bond distance for complex **3** was incorrectly quoted as 1.363(4) Å in the Results and discussion section on page 2026. The correct C–O bond distance in complex **3** is 1.158(6) Å, while the one quoted in the manuscript of 1.363(4) Å is the C–O distance in the U–O–C(*ipso*).

The Royal Society of Chemistry apologises for these errors and any consequent inconvenience to authors and readers.

## Supplementary Material

