# Supplementary material for: Isolation of C1 through C4 derivatives from CO using heteroleptic uranium(iii) metallocene aryloxide complexes
Source: Chem Sci. 2023 Feb 6;14(8):2024–32. doi: 10.1039/d2sc06375a (PMC9945253; doi:10.1039/d2sc06375a)
Supplement: SC-014-D2SC06375A-s001 [file SC-014-D2SC06375A-s001.pdf]

Electronic Supplementary Material (ESI) for Chemical Science.
This journal is © The Royal Society of Chemistry 2023

Supporting Information for

Isolation of C1 through C4 Derivatives from CO using Heteroleptic Uranium(III) Metallocene Aryloxide Complexes

Robert J. Ward,[a] Iker Del Rosal,[b] Steven P. Kelley,[a] Laurent Maron,[b]* and Justin R. Walensky[a]*

[a] Department of Chemistry, University of Missouri, Columbia, MO 65211 USA

[b] Universite de Toulouse and CNRS, INSA, UPS, CNRS, UMR, UMR 5215, LPCNO
135 Avenue de Rangueil, 31077 Toulouse (France)

Table of Contents

General considerations……………………………………………………S2

Synthesis of [$(C_5Me_5)_2$(2,6-$^tBu_2$-4-MeC_6H_2-O)U(CO)], 3……………………S3

Figure S1. ^{1}H NMR spectrum of **3** in C_6D_6……………………………………S3

Figure S2. Infrared (KBr) spectrum of **3**……………………………………S4

Figure S3. UV-vis-nIR spectrum of **3**……………………………………S4

Synthesis of [{$(C_5Me_5)_2$(MesO)U}$_2$(μ_2-OCCO)], 4……………………………S5

Figure S4. ^{1}H NMR spectrum of **4** in C_6D_6……………………………………S5

Figure S5. ^{13}C labeled ^{13}C NMR spectrum of **4** in C_6D_6…………………………S6

Figure S6. ^{13}C labeled DEPT 135 spectrum of **4** in C_6D_6…………………………S6

Figure S7. Infrared (KBr) spectrum of **4**……………………………………S7

Synthesis of [{$(C_5Me_5)_2$(OMes)U}$_2$(μ_2:κ^2:κ^1-C_3O_3)], 5…………………S7

Figure S8. ^{1}H NMR spectrum of **5** in C_6D_6……………………………………S8

Figure S9. Infrared (KBr) spectrum of **5**……………………………………S9

Synthesis of [{$(C_5Me_5)_2$(MesO)U}$_2$(μ-κ^2-(O,O)-O_2C(C=C=O)CO_2] 6……………S9

Figure S10. ^{1}H NMR spectrum of **6** in C_6D_6……………………………………S10

Figure S11. Infrared (KBr) spectrum of **6**……………………………………S11

Synthesis of [{$(C_5Me_5)_2$U}$_2$(OC(CPh_2)C(=O)CO)], 7, and [$(C_5Me_5)_2$U$(OMes)_2$, 8……S11

Figure S12. ^{1}H NMR spectrum of **7** in C_6D_6……………………………………S11

Figure S13. Infrared (KBr) spectrum of **7**……………………………………S12

Independent synthesis of [$(C_5Me_5)_2U(OMes)_2$], 8……………..………………………………S12

Figure S14. ^{1}H NMR spectrum of **8** in C_6D_6………..………………………………………S12

Figure S15. Infrared (KBr) spectrum of **8**……………………………………………...……S13

[$(C_5Me_5)_2$(OMes)U{μ-κ^2-(O,O)-O_2CC(O)(SO)-κ^2-(O,O)}U(OMes)$(C_5Me_5)_2$], 9………………....S13

Figure S16. ^{1}H NMR spectrum of **9** in C_6D_6……………………………………………..….S14

Figure S17. Infrared (KBr) spectrum of **9**………………………………………………...S15

Crystal structure refinement details……………………….…………………………..S15

Table S1. X-ray crystallographic details for complexes **3-6**…………………………..….S17

Table S2. X-ray crystallographic details for complexes **7-9**…………………………..….S18

Computational details……………………………………………………………………S19

Table S3. Selected geometrical parameters comparison between the optimized structures at different spin states and the experimental one for complex **3**……………………………S19

Figure S18. 3D representation of the SOMO-2 of complex **3** showing the major contribution to the backbonding in CO……………………………………………………………………..S20

Figure S19. 3D representation of the HOMO-4 of complex **3** showing the interaction of the $(C_5Me_5)^{1-}$ ligand which has a minor contribution to the backbonding in CO……………………..S20

Figure S20. Energetic difference between carbon bound versus oxygen bound CO in complex **3**..S21

Figure S21. Effect of the aryloxide position to the stability of complex **4**……………….……S21

References……...……………………………………………………………………..S22

General consideration. All syntheses were carried out under an inert atmosphere of nitrogen using standard Schlenk and glovebox techniques. All solvents were purchased anhydrous, stored over activated 4 Å molecular sieves, and sparged with nitrogen prior to use. All commercially available reactants were purchased from suppliers and used without further purification. $(C_5Me_5)_2$(2,6-^{t}Bu$_2$-4-MeC$_6$H$_2$-O)U, **1**,[1] $(C_5Me_5)_2$(MesO)U(THF), **2**,[2] diphenylketene,[3] and DABSO,[4] were synthesized as previously described. Benzene-d_6 was dried over molecular sieves and degassed with three cycles of freeze-pump-thaw. All ^{1}H and ^{13}C NMR spectra were collected either on a Bruker Avance III 500 or 600 MHZ spectrometer. ^{1}H and ^{13}C NMR chemical shifts were referenced internally to the residue solvent peak at 7.16 ppm (C_6D_5H) and 128.06 ($^{13}C_6D_6$). Infrared spectra were recorded as a KBr pellets on Perkin-Elmer Spectrum One FT-IR spectrometer. Elemental analysis was performed on a Carlo Erba 1108 elemental analyzer, outfitted with an A to D converter for analysis using Eager Xperience software.

Caution! *Uranium-238 (depleted uranium) is an alpha-emitting radiometal with half-lives of 4.47 × 10^9 years. All work was carried out in a radiological laboratory with appropriate personal protective and counting equipment.*

Synthesis of [$(C_5Me_5)_2$(2,6-^{t}Bu_2-4-MeC_6H_2-O)U(CO)], 3: To a saturated solution of **1** in hexamethyldisiloxane solution in a 100 mL Straus flask, carbon monoxide was added, and the flask was sealed. The solution changes from brown to green-brown color. The solution is heated to 60 °C, for an hour, and slowly brought to 6 °C, to yield dark brown crystals of **3**. ^{1}H NMR (C_6D_6, 298 K): -5.61 (s, 30H, $C_5(CH_3)_5$), -3.75 (bs, 18H, o-Ar-C(CH_3)$_3$), 5.57 (s, 3H, p-Ar-CH_3), 12.46 (bs, 2H, m-Ar-**H**). IR (KBr, cm^{-1}): 2928.9 (m), 2919.2 (m), 2856.1 (m), 1904.4 (m C☰O), 1392.8 (m), 1384.6 (s), 1117.5 (bs), 826.3 (w), 809.0 (w), 527.9 (w), 419.4 (w). IR (C_6D_6, cm^{-1}): 1893 (C☰O). IR (C_6D_6, cm^{-1}): 1852.6 (^{13}C☰O). Crystals of **3** were dissolved in deuterated benzene, without a carbon monoxide atmosphere. After 15 hrs ~60% of **3** had converted back to **1**. Complete conversion of **3** to **1** was achieved after 20 minutes under reduced pressure.

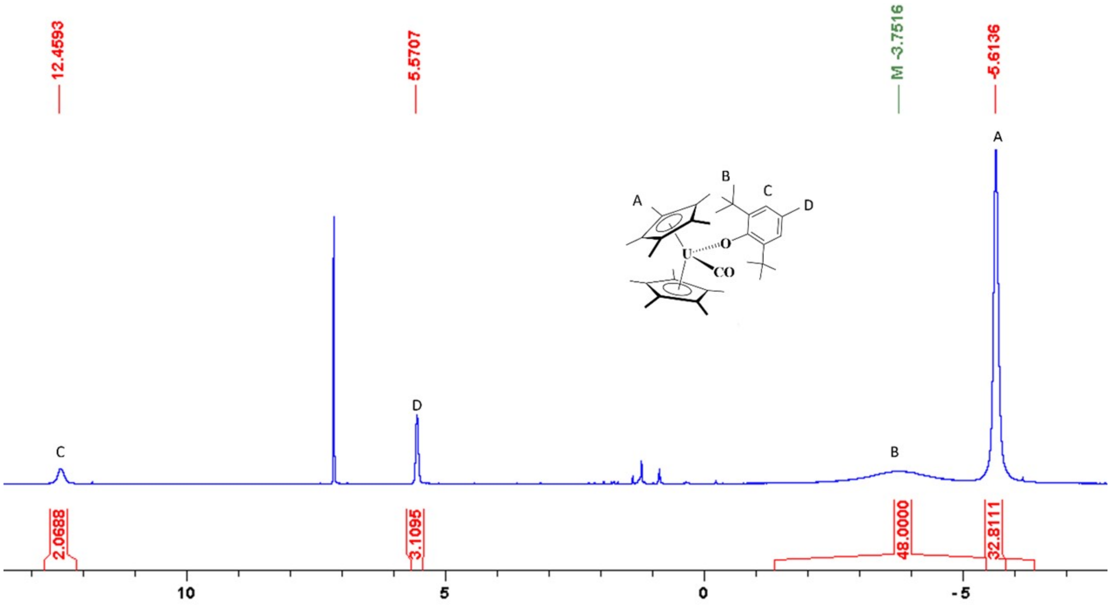

Figure S1. ^{1}H NMR spectrum of **3** in C_6D_6.

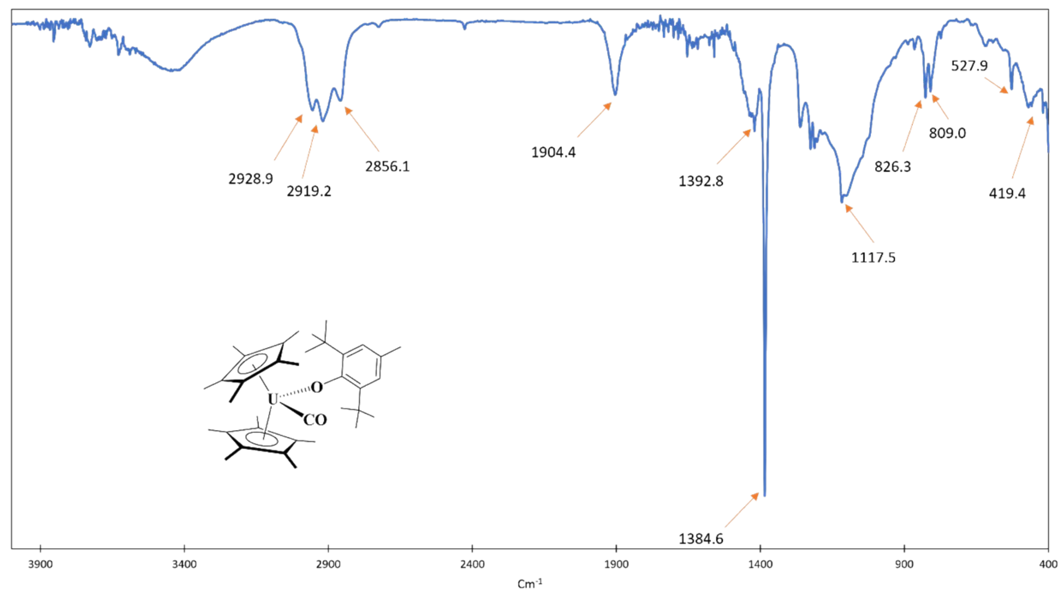

Figure S2. Infrared (KBr) spectrum of **3**.

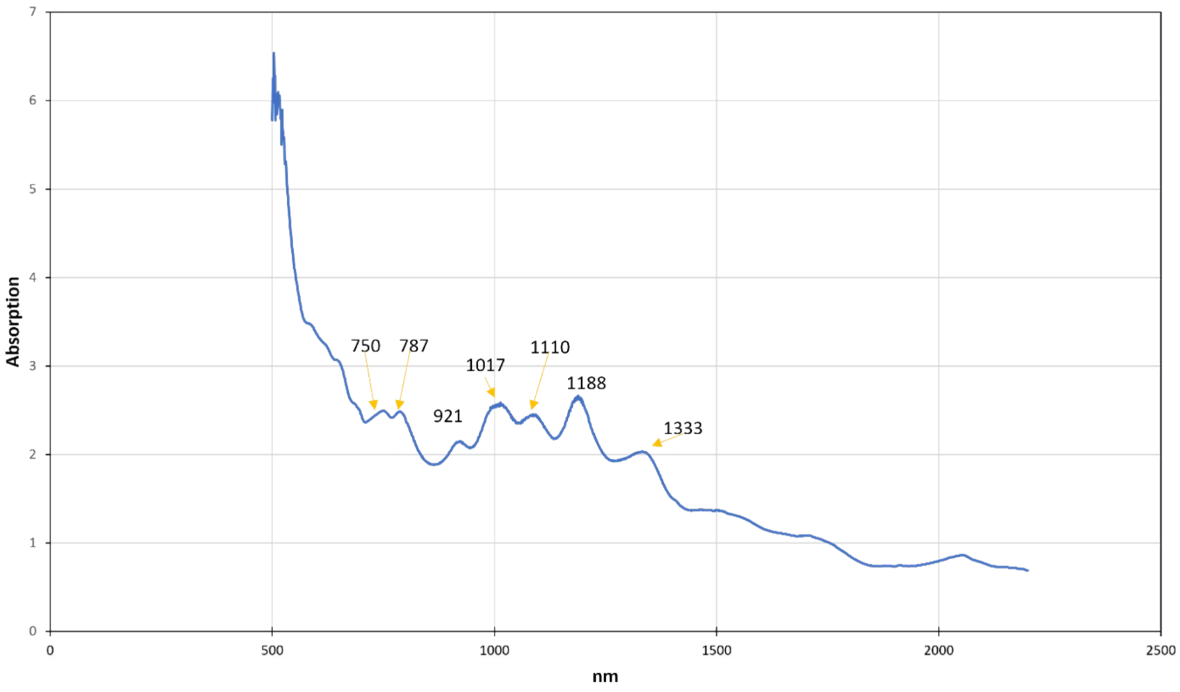

Figure S3. UV-vis-nIR spectrum of **3** in toluene.

Synthesis of [{$(C_5Me_5)_2$(MesO)U}$_2$(μ_2-OCCO)], 4. To a dark green degassed pentane (20 mL) solution of [$(C_5Me_5)_2$(MesO)U(THF)], Mes = 2,4,6-$Me_3C_6H_2$ (**2**) (0.417 g, 0.583 mmol), in a 100 mL storage flask, carbon monoxide was added (1 atm). The solution immediately turned dark red-black, and was allowed to stir for twelve hours. After stirring, carbon monoxide was removed and the red suspension was filtered to yield a red powder, which was recrystalized from toluene at -45 °C yielding dark red needles (0.249 g, 0.185 mmol, 89%). ^{1}H NMR (C_6D_6, 298 K): δ -30.98 (s, 6H, o-Ar-C**H**$_3$), -17.76 (s, 6H, o-Ar-C**H**$_3$), 0.05 (s, 6H, p-Ar-C**H**$_3$), 1.07 (s, 60H, C_5(C**H**$_3$)$_5$), 1.48 (s, 2H, m-Ar-**H**), 2.54 (s, 2H, m-Ar-**H**). IR (KBr, cm^{-1}): 2962.6 (m), 2908.6 (s), 2857.0 (s), 2009.9 (wb C≡C), 1473.8 (m), 1384.2 (s), 1341.7 (s), 1236.6 (s), 1157.6 (m), 833.1 (s) 729.0 (w), 527.0 (w). Anal. Calcd. theory (found) for $C_{60}H_{82}U_2O_4$, C 53.65 (54.02%), H 6.15 (5.68%).

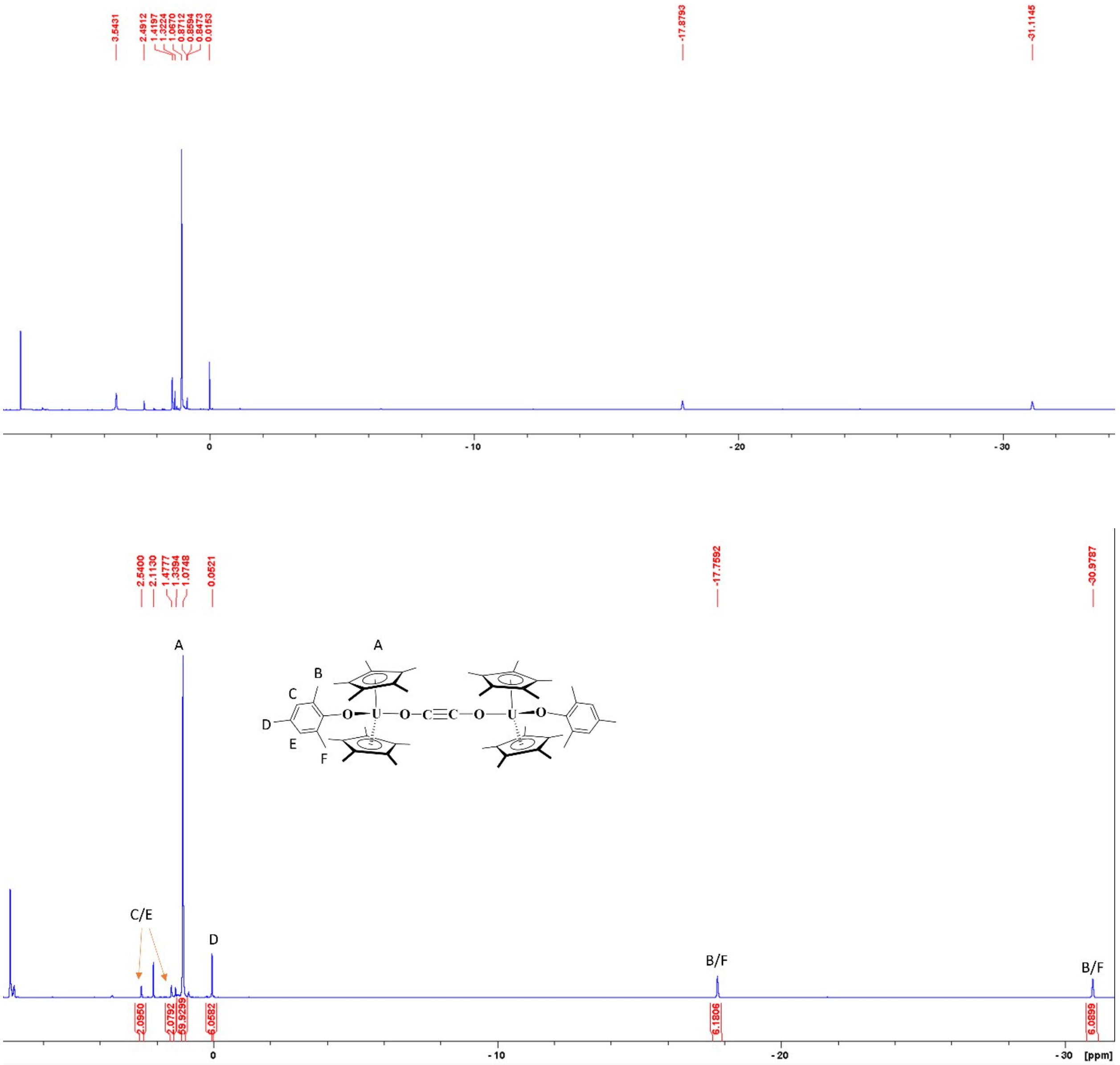

Figure S4. Top: crude ^{1}H NMR spectrum of **4** in C_6D_6. Bottom: ^{1}H NMR spectrum of **4** isolated in C_6D_6.

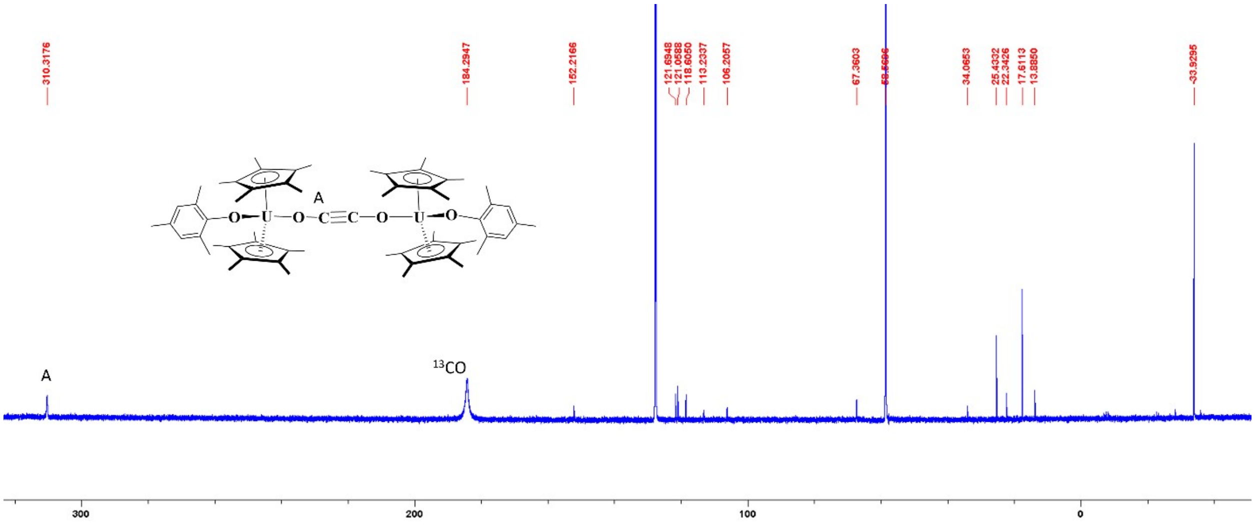

Figure S5. ^{13}C labeled ^{13}C NMR spectrum of **4** in C_6D_6.

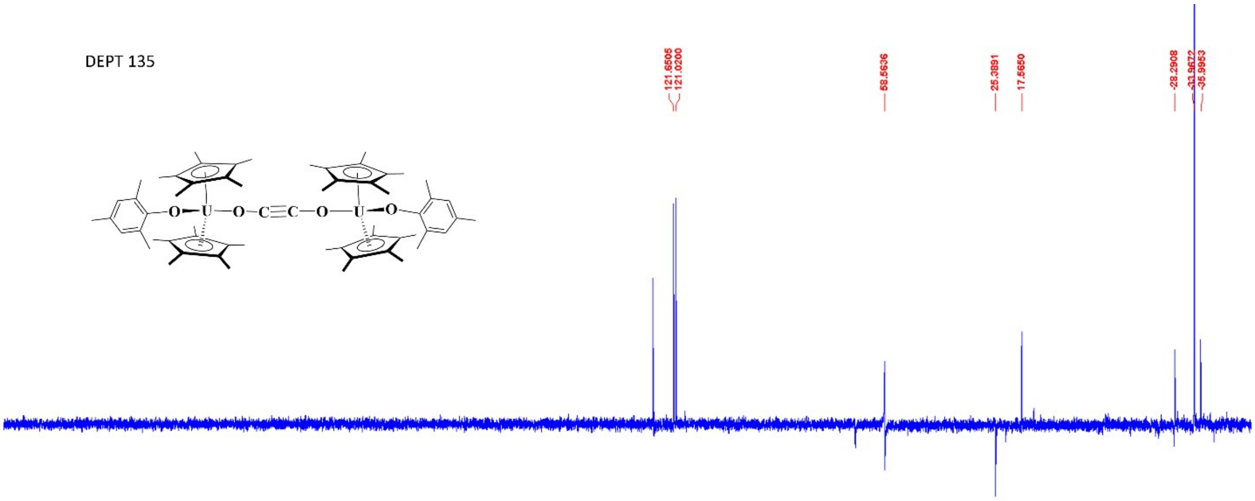

Figure S6. ^{13}C labeled DEPT 135 spectrum of **4** in C_6D_6.

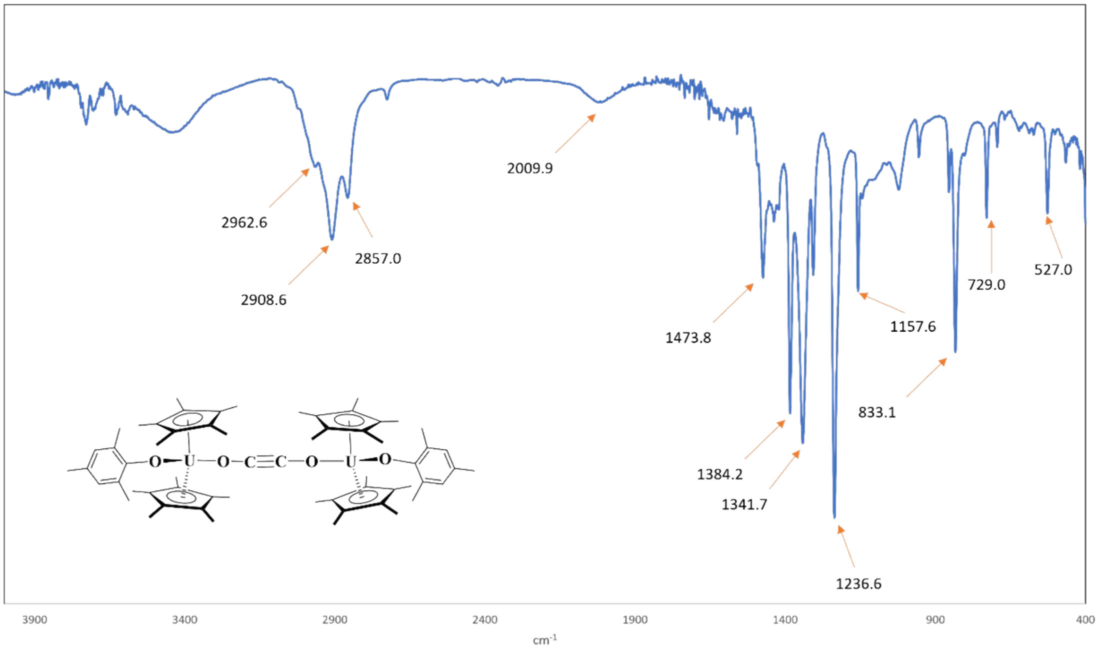

Figure S7. Infrared (KBr) spectrum of **4**.

Synthesis of [{(C_5Me_5)$_2$(OMes)U}$_2$(μ_2:κ^2:κ^1-C_3O_3)], 5. In a J. Young NMR tube, a solution of [(C_5Me_5)$_2$(MesO)U(THF)], **2**, (21.9 mg, 0.0306 mmol) and deuterated benzene (2 mL), was treated with carbon monoxide (1 atm). After heating to 80 °C, for 3 days the initially formed **4**, had disappeared from the NMR spectrum. Volatiles were removed under reduced pressure and the product was recrystallized in pentane to yield **5** (10.4 mg, 0.0076 mmol, 50%). ^{1}H NMR (C_6D_6, 298 K): δ -35.31 (s, 3H o-Ar-C**H**$_3$), -25.52 (s, 3H o-Ar-C**H**$_3$), -21.13 (s, 3H o-Ar-C**H**$_3$), -12.40 (s, 3H o-Ar-C**H**$_3$), -0.37 (s, 30H C_5(C**H**$_3$)$_5$), -0.13 (s, 30H C_5(C**H**$_3$)$_5$), 3.02 (s, 1H m-Ar-**H**), 3.18 (s, 1H m-Ar-**H**), 4.74 (s, 3H, p-Ar-C**H**$_3$), 8.72 (s, 1H m-Ar-**H**), 8.83 (s, 1H m-Ar-**H**), 9.50 (s, 3H p-Ar-C**H**$_3$). IR (KBr, cm^{-1}): 2933.2 (m), 2911.5 (s), 2857.5 (s), 2203.3 (wb), 2065.4 (s C=O), 1473.3 (m), 1411.2 (m), 1384.2 (s), 1219.8 (s), 1166.6 (m), 1018.7 (w), 832.1 (m), 525.5 (w).). Anal. Calcd. theory (found) for $C_{61}H_{82}U_2O_5$, C 53.43 (53.19%), H 6.03 (6.42%).

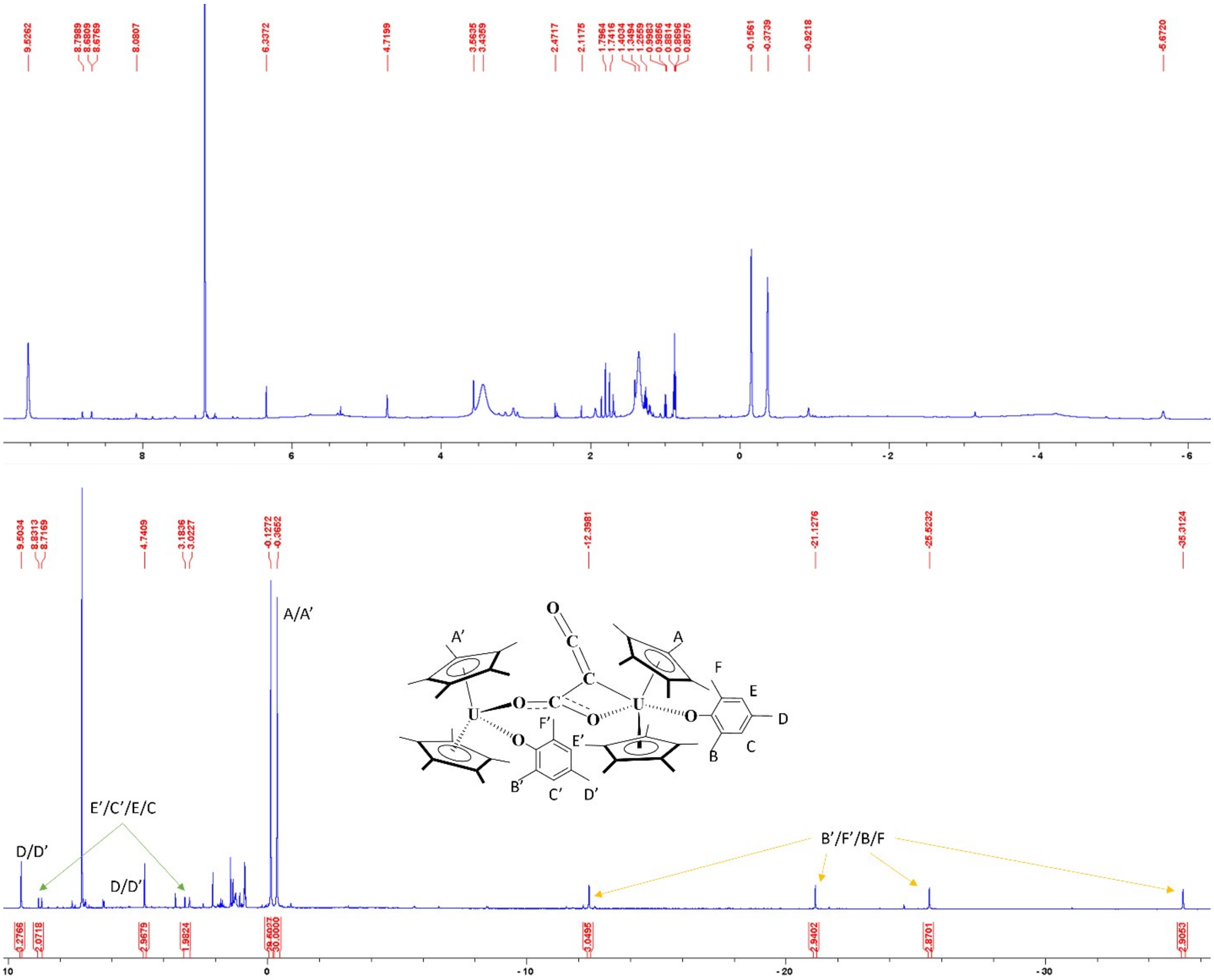

Figure S8. Top: crude ^{1}H NMR spectrum of **5** in C_6D_6. Bottom: ^{1}H NMR spectrum of **5** isolated in C_6D_6.

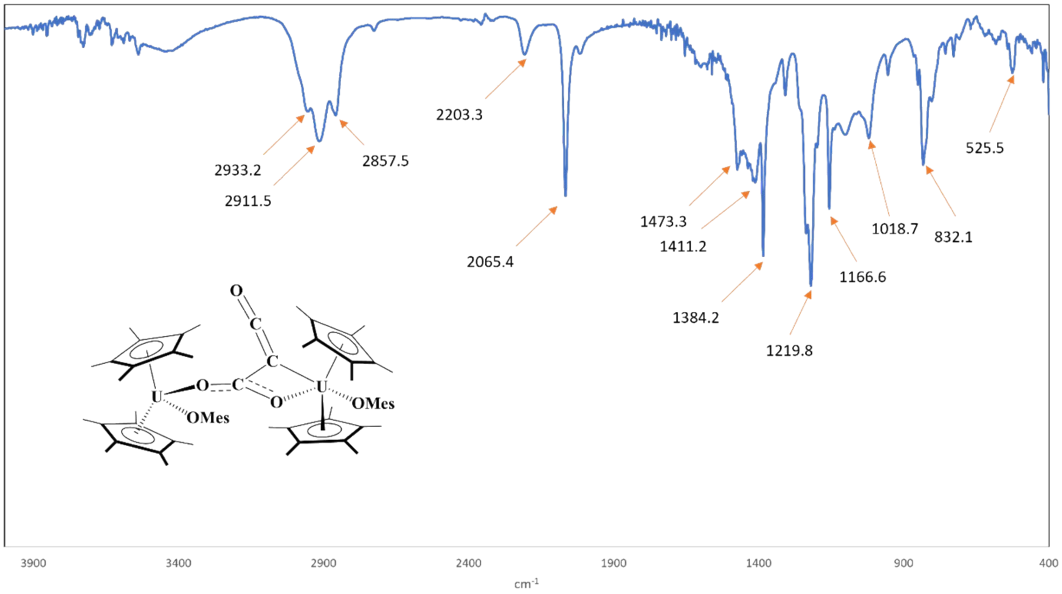

Figure S9. Infrared (KBr) spectrum of **5**.

Synthesis of [{(C_5Me_5)$_2$(MesO)U}$_2$(μ-κ^2-(O,O)-O_2C(C=C=O)CO_2] 6: In a J. Young NMR tube, a solution of **5** (53.4 mg, 0.0389 mmol) and C_6D_6 (2 mL), was treated with CO_2 (1 atm). After sitting at room temperature for 12 hours volatiles were removed and the red product was recrystallized from pentane yielding red crystals (14.4 mg, 0.0102 mmol, 26%). ^{1}H NMR (C_6D_6, 298 K): δ -0.89 (s, 60H, C_5(C**H**$_3$)$_5$), 17.44 (s, 6H, Ar-C**H**$_3$), 27.19 (s, 2H, m-Ar-**H**), 33.49 (s, 2H, m-Ar-**H**), 44.24 (s, 6H, Ar-C**H**$_3$). Two mesityl methyl groups were not observed. IR (KBr, cm^{-1}): 2961.2 (m), 2911.5 (m), 2855.6 (w), 1560.6 (mb), 1473.8 (s), 1384.2 (s), 1220.2 (m), 1154.7 (m), 1019.2 (w), 940.1 (wb), 806.1 (m), 801.3 (m). Anal. Calcd. theory (found) for $C_{62}H_{82}U_2O_7$, C 52.61 (52.74%), H 5.84 (5.48%).

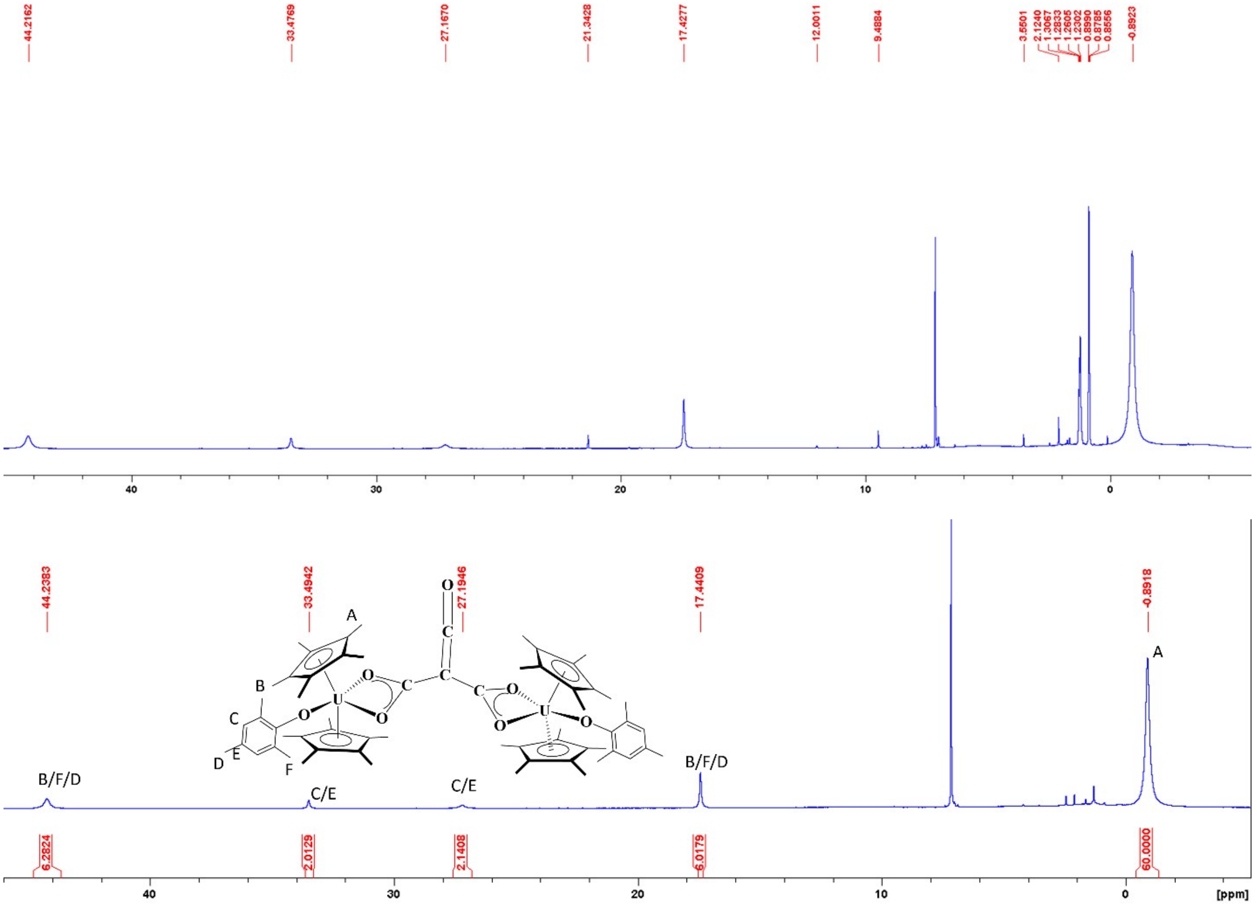

Figure S10. Top: crude ¹H NMR spectrum of **6** in C_6D_6. Bottom: ¹H NMR spectrum of **6** isolated in C_6D_6.

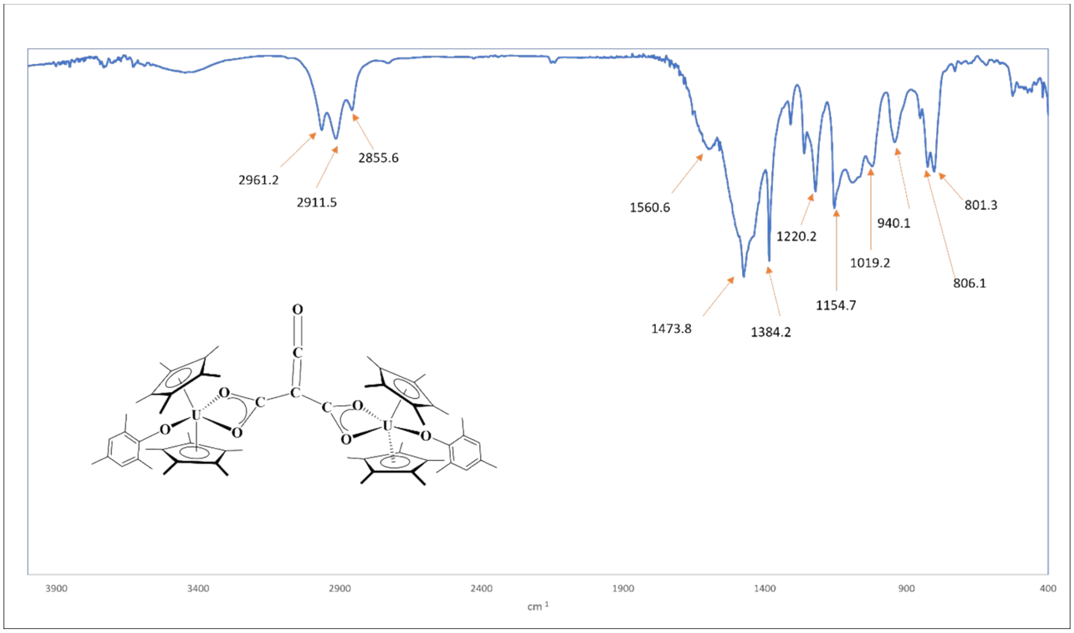

Figure S11. Infrared (KBr) spectrum of **6**.

Synthesis of [{(C_5Me_5)$_2$U}$_2$(OC(CPh$_2$)C(=O)CO)], 7, and [(C_5Me_5)$_2$U(OMes)$_2$, 8. To a toluene solution (10 mL) of **4** (0.1180 g, 0.0879 mmol), diphenylketene (0.0171 g, 0.0880 mmol) was added. The solution was stirred overnight, and filltered. The solution stored at room temperature. After two days X-ray quality crystals of **7** (0.0140 g, 0.0092 mmol, 21%), were isolated. ^{1}H NMR (CD_2Cl_2, 298 K): δ -11.41 (s, 8H, o-Ph-**H**), 3.54 (d, 8H, m-Ph-**H**), 5.06 (t, 4H, p-Ph-**H**), 5.47 (s, 60H, $C_5(C\mathbf{H}_3)_5$). IR (KBr, cm^{-1}): 2939.9 (m), 2914.9 (m), 2855.1 (m), 1751.5 (m) (C=O), 1574.6 (s), 1437.7 (mb), 1384.6 (s), 1355.2 (s), 1109.4 (m), 925.7 (m), 897.2 (w), 695.7 (w). Anal. Calcd. theory (found) for $C_{72}H_{80}U_2O_6$, C 56.99 (57.00%), H 5.31 (5.54%).

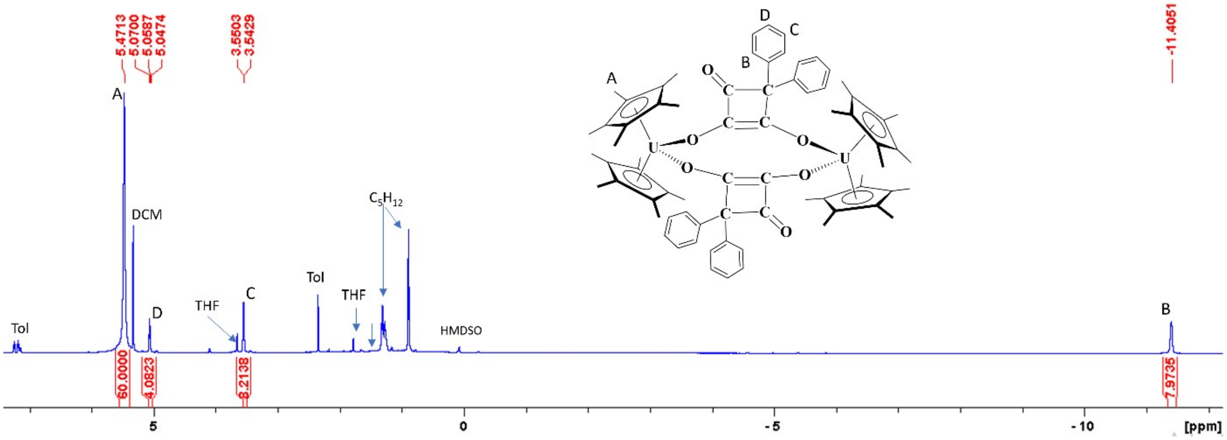

Figure S12. ^{1}H NMR spectrum of **7** in C_6D_6.

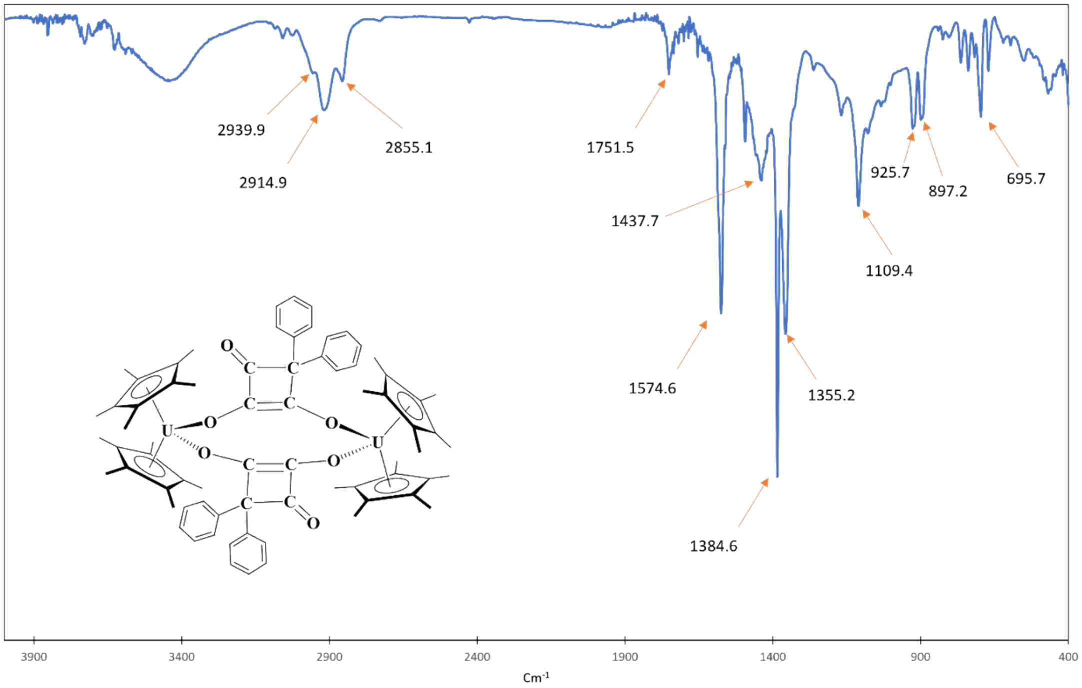

Figure S13. Infrared (KBr) spectrum of **7**.

Independent synthesis of [$(C_5Me_5)_2U(OMes)_2$], 8. [$(C_5Me_5)_2UCl_2$] (78.5 mg, 0.1355 mmol) was dissolved in THF (10 mL), to which KOMes (51.9 mg, 0.2978 mmol) was added. The solution immediately turned from red to orange, and became cloudy. After 20 minutes the volatiles were removed under vacuum, and the solid was redissolved in 20 mL of pentane. After stirring for ten minutes the pentane solution was filled through diatomaceous earth, and cooled to -45 °C to yield orange crystals of **8** (38.5 mg, 0.0494 mmol, 36.5%). ^{1}H NMR (C_6D_6, 298 K): δ -8.79 (s, 6H, o-Ar-C**H**$_3$), -3.16 (s, 6H, o-Ar-C**H**$_3$), 2.47 (s, 6H, p-Ar-C**H**$_3$), 3.59 (s, 30H, C_5(C**H**$_3$)$_5$), 6.76 (s, 2H, Ar-**H**), 7.88 (s, 2H, Ar-**H**). IR (KBr, cm^{-1}): 2972.7 (m), 2912.9 (s), 2855.1 (s), 1473.8 (s), 1384.2 (m), 1306.1 (s), 1232.8 (s), 1218.5 (s), 1156.1 (s), 951.2 (w), 852.4 (m), 833.6 (m), 821.5 (s), 729.0 (w), 520.7 (m). Anal. Calcd. theory (found) for $C_{38}H_{52}UO_2$, C 58.60 (58.82%), H 6.73 (6.94%).

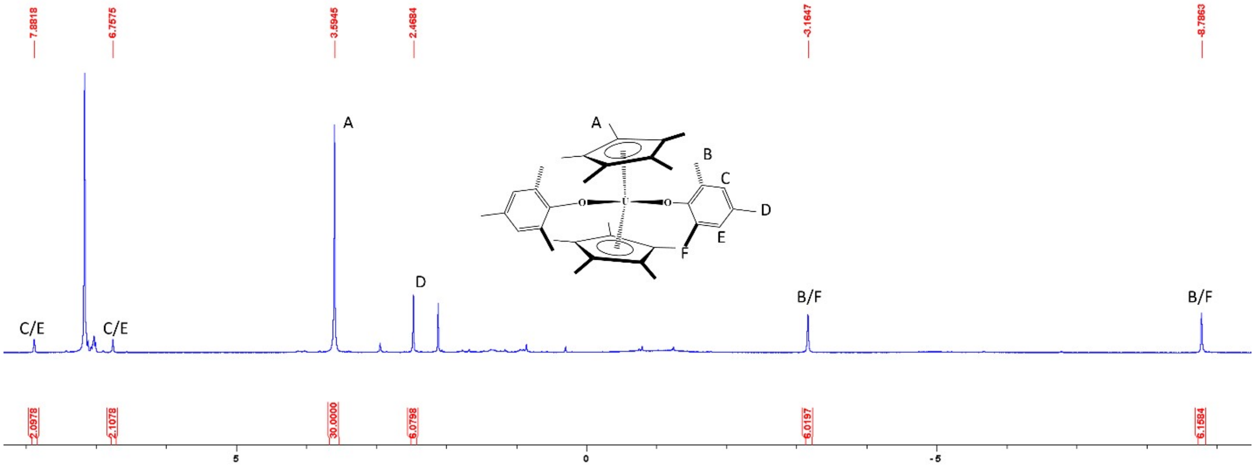

Figure S14. ^{1}H NMR spectrum of **8** in C_6D_6.

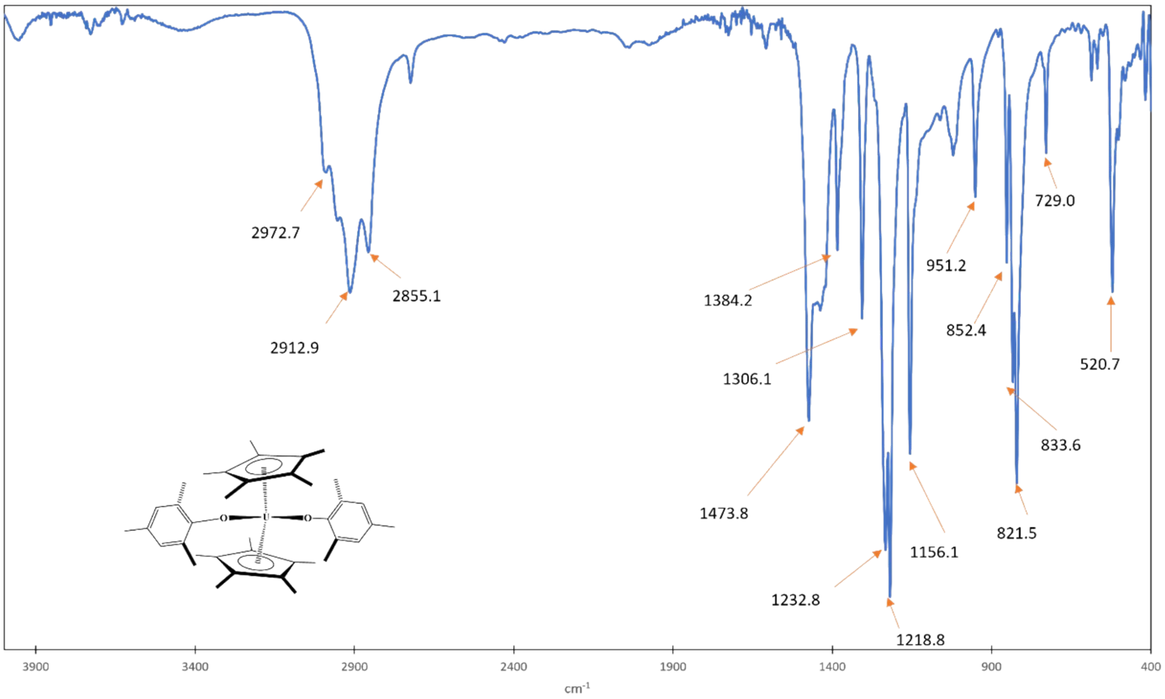

Figure S15. Infrared (KBr) spectrum of **8**.

[(C_5Me_5)$_2$(OMes)U{μ-κ^2-(O,O)-O_2CC(O)(SO)-κ^2-(O,O)}U(OMes)(C_5Me_5)$_2$], 9: A -45 °C solution of **4** (99.6 mg, 0.0741 mmol), in toluene (8 mL) was added to a suspension of DABSO (8.9 mg, 0.0370 mmol), in -45 °C toluene (5 mL). The solution was allowed to slowly warm to room temperature and after two hours volatiles were removed under vacuum. The dark powder was rinsed with pentane (2 x 3 mL). The solid was recrystallized from a saturated toluene solution at -14 °C to yield small dark red needles of **9** (31.0 mg, 0.0220 mmol, 30%). ^{1}H NMR (C_6D_6, 298 K): -4.30 (s, 30H, C_5(C**H**$_3$)$_5$), -3.35 (s, 3H, p-Ar-C**H**$_3$), 3.22 (s, 30H, C_5(C**H**$_3$)$_5$), 7.60 (s, 3H, p-Ar-C**H**$_3$), 13.25 (s, 3H, o-Ar-C**H**$_3$), 17.72 (s, 3H, o-Ar-C**H**$_3$), 21.29 (s, 1H, m-Ar-**H**), 25.79 (s, 1H, m-Ar-**H**), 26.79 (s, 3H, o-Ar-C**H**$_3$), 27.64 (s, 1H, m-Ar-**H**), 32.83 (s, 1H, m-Ar-**H**), 39.55 (s, 3H, o-Ar-C**H**$_3$). IR (KBr, cm^{-1}): 2906.2 (s), 2856.5 (s), 1544.7 (m), 1540.8 (m), 1473.4 (s), 1407.3 (s), 1220.2 (s), 1157.1 (s), 889.0 (m), 825.4 (s), 728.5 (w), 524.5 (m). Anal. Calcd. theory (found) for $C_{60}H_{82}U_2O_6S$, C 51.20 (51.00%), H 5.87 (5.48%).

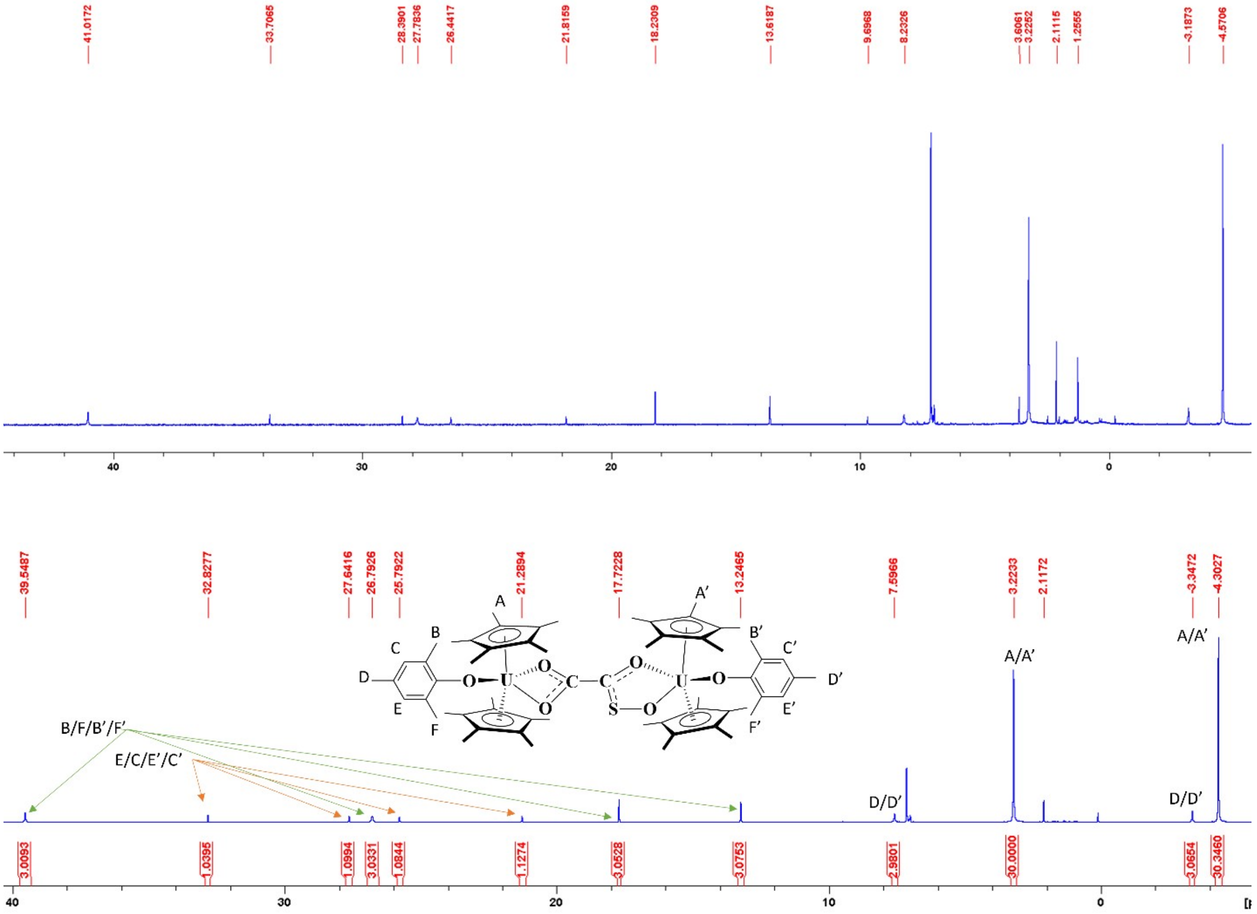

Figure S16. Top: crude ¹H NMR spectrum of **9** in C_6D_6. Bottom: ¹H NMR spectrum of **9** isolated in C_6D_6.

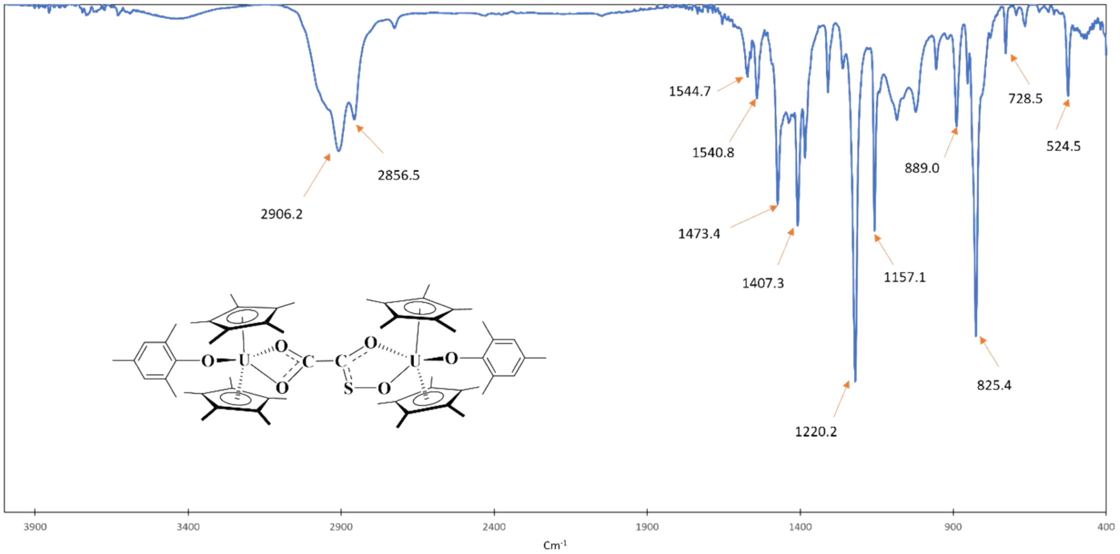

Figure S17. Infrared (KBr) spectrum of **9**.

Crystal Structure Refinements:

Single crystal X-ray diffraction (SCXRD) data for **3**, **6**, and **9** were collected on a Bruker X8 Prospector diffractometer equipped with an APEX II CCD (Bruker AXS, Madison, WI, USA) area detector using Cu Kα radiation from a microfocus source (λ = 1.54178 Å; beam power = 45 kV, 0.65 mA). Data for **4**, **5**, **7**, and **8** were collected on a Bruker SMART diffractometer equipped with an APEX II detector using Mo Kα radiation from a sealed source with focusing optics (λ = 0.71073 Å; beam power = 50 kV, 30 mA). Crystals were cooled to the collection temperatures under streams of cold N_2 gas using Cryostream 700 cryostats (Oxford Cryosystems, Oxford, UK). Hemispheres of unique data were collected out to the desired resolution limit using strategies of 0.5^o scans about the omega and phi axes. Unit cell determination, data collection, data reduction, absorption correction and scaling, and space group determination were performed using the Apex3 software suite.[5]

Crystal structures were solved using either direct methods as implemented in SHELXS v.2013.1 [6]or an iterative dual space approach as implemented in SHELXT. .[7] Full occupancy non-hydrogen atoms were located from the difference map and refined anisotropically by full matrix least squares refinement against F^2 using SHELXL[8] Olex2 was used as a graphical interface for model building and structure visualization.

When structure **4** was refined at full occupancy, the thermal ellipsoids of the ethynediolate ligand atoms were anomalously small, and the difference map contained a single large peak near the U atom and numerous smaller peaks surrounding the ethynediolate ligand. The difference between the large peak near the U atom and the nearest ethynediolate C atom is very close to the published U-I distance in complexes such as $U(Cp^*)_2I_2$. The largest difference map peak and a smaller peak overlapping with C30 were modeled as partial occupancy U and I atoms. The occupancies of the major U atom and the ethynediolate atoms were refined as a single parameter. The occupancies of both U atoms were constrained to sum to 100%, and their anisotropic displacement parameters were constrained to be equal. Unrealistically short bonds between the

two symmetry-equivalent I atom positions were excluded from the model using a negative SHELX PART number. Because only one of the two U atoms per unit cell can possibly be bonded to the I atom, the occupancy of I was set to one half of the occupancy of the minor U atom. The occupancy of the major component refined to 94.7(3)%. The remaining 5.3 % of crystallographic sites are occupied by either $UI(C_5Me_5)_2(OMes)$ or compound **1**. I^- is attributed to contamination from $[(C_5Me_5)_2UI(THF)]$, the precursor to **1**. The I atom had to be refined isotropically and has an unusually large U_{iso} value, both of which are likely caused by the imprecision in the estimate of the near-zero occupancy of this atom.

The final refinement of **6** has severe difference map artifacts, numerous anomalous anisotropic displacement parameters for most light atoms, and unusually high R indices and merging R values for equivalent reflections. Diffraction photographs show that low-index peaks are sharp but become very diffuse at higher angles. Peaks in *hk*0 zone at high *k* indices have diffuse diffraction along the *h* direction. This can occur if the crystal is composed of slightly misaligned stacks of plates, which was consistent with the visual appearance of the crystals under the microscope. It could also indicate a loss of order in the *a* direction, which is consistent with the presence of regions of disordered matter that separate layers along *a*. The most satisfactory model was obtained by integrating the data as a single domain and using restraints where appropriate to prevent these artifacts from affecting the model. C_5Me_5 and mesityl ligands had their chemically equivalent C-C distances restrained to be equal within +/- 0.02 Å. One C_5Me_5 ligand could be modeled as disordered over two positions related by rotation about the U-centroid axis; the occupancies were fixed 60% and 40%, and the minor part was refined isotropically. Restraints for rigid bond behavior were applied to the anisotropic displacement parameters of all atoms.[9] The structure contained solvent accessible void space after modeling of all interpretable difference map peaks. PLATON SQUEEZE was used to estimate the contents of these voids and remove their contribution from the structure factors.[10] The analysis found 497 e^- of disordered matter distributed across 1610 $Å^3$ of void space per unit cell, which corresponds to approximately 1.5 pentane molecules per molecule of the main moiety.

The data collection of **9** was truncated to a resolution of 1.0 Å. Repeated attempts at data collection on crystals of this compound failed to produce stronger high angle diffraction. The weak diffraction is due to a combination of the size of the unit cell, the small size of the crystal, and the presence of large volumes of disordered matter. The two disordered Cp* rings had their chemically equivalent C-C aromatic distances and 1,3 C---C nonbonding distances restrained to be equal; separate restraints were applied for each ring. The crystal contains channels of solvent-accessible void space along the crystallographic rotoinversion axes. Disordered mattered in these voids was treated using PLATON SQUEEZE which found 1,334 e^- of disordered matter per unit cell, corresponding to approximately 1.5 toluene molecules per molecule of **9**.

Table S1. X-ray crystallographic details for complexes **3-6**.

	**3**	**4**	**5**	**6**
**CCDC deposit number**	2220200	2220201	2220202	2220203
**Chemical formula**	$C_{36}H_{53}O_2U$	$C_{60}H_{82}O_4U_2$[a]	$C_{61}H_{82}O_5U_2 \cdot C_5H_{12}$	$C_{62}H_{82}O_7U_2 \cdot 1.5(C_5H_{12})$
**Formula weight (g/mol)**	755.81	1343.32	1443.47	1513.47
**Crystal habit/color**	Needle/Brown	Prism/Orange	Prism/Orange	Plate/Red
**Temperature (K)**	150(2)	100(2)	150(2)	150(2)
**Space group**	$P2_12_12_1$	P-1	$P2_1/c$	$C2/c$
**Crystal system**	Orthorhombic	Triclinic	Monoclinic	Monoclinic
**Volume (Å)**	3270.95(15)	1387.6(4)	6034.2(9)	12497.8(8)
**a (Å)**	10.7675(3)	9.4109(16)	10.5339(9)	43.0997(15)
**b (Å)**	17.3155(4)	10.8091(17)	26.165(2)	14.4756(6)
**c (Å)**	17.5438(5)	14.131(2)	21.8935(18)	20.4007(7)
**α (deg)**	90	94.671(5)	90	90
**β (deg)**	90	103.949(5)	90.283(2)	100.911(3)
**γ (deg)**	90	92.409(6)	90	90
**Z**	4	1	4	8
**Calculated density (g/cm³)**	1.535	1.610	1.589	1.609
**Absorption coefficient (mm⁻¹)**	14.171	5.888	5.408	14.878
**Final *R* indices [$I > 2\sigma(I)$]**	R = 0.0140	R = 0.0225	R = 0.0460	R = 0.1225
	R_w = 0.0332	R_w = 0.0444	R_w = 0.0865	R_w = 0.2819

[a]Formula excludes substitutional disordered species which are included in the theoretical density and absorption coefficient.

Table S2. X-ray crystallographic details for complexes **7-9**.

	**7**	**8**	**9**
**CCDC deposit number**	2220204	2220205	2220206
**Chemical formula**	$C_{72}H_{80}O_6U_2$•2(C_7H_8)	$C_{38}H_{52}O_2U$	$C_{60}H_{92}O_6SU_2$·1.5(C_7H_8)
**Formula weight (g/mol)**	1701.69	778.82	1545.57
**Crystal habit/color**	Rod/Red	Plate/Red	Prism/Red
**Temperature (K)**	220(2)	150(2)	150(2)
**Space group**	$P2_1/n$	$P2_1/c$	*R*-3
**Crystal system**	Monoclinic	Monoclinic	Trigonal
**Volume (Å)**	3552.3(6)	3309.5(18)	28601.9(19)
**a (Å)**	17.9293(18)	13.266(4)	56.6846(17)
**b (Å)**	10.2778(10)	16.193(5)	56.6846(17)
**c (Å)**	19.647(2)	15.544(5)	10.2786(3)
**α (deg)**	90	90	90
**β (deg)**	101.1333(17)	97.637(4)	90
**γ (deg)**	90	90	120
**Z**	2	4	18
**Calculated density (g/cm^3)**	1.591	1.563	1.615
**Absorption coefficient (mm^{-1})**	4.608	4.935	14.927
**Final *R* indices [$I > 2\sigma(I)$]**	R = 0.0315	R = 0.0333	R = 0.0419
	R_w = 0.0536	R_w = 0.0638	R_w = 0.0972

Computational details.

All DFT calculations were carried out with the Gaussian 09 suite of programs.[11] Geometries were fully optimized in gas phase without symmetry constraints, employing the B3PW91 functional.[12, 13] The nature of the extrema was verified by analytical frequency calculations. The calculation of electronic energies and enthalpies of the extrema of the potential energy surface (minima and transition states) were performed at the same level of theory as the geometry optimizations. IRC calculations were performed to confirm the connections of the optimized transition states. Uranium was treated with a small core effective core potential (60 MWB), associated with its adapted basis set.[14, 15] For the other elements (H, C, S and O), Pople's double-ζ basis set 6-31G(d,p) was used.[16-18]The electronic charges (at the DFT level) were computed using the natural population analysis (NPA) technique.[19]

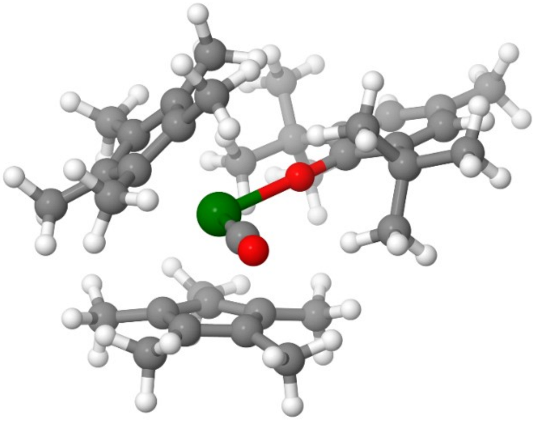

Multiplicity	doublet	quartet	sextet	Exp.
$\Delta_r H$ (kcal/mol)	13.8	0.0	55.5	-
distances (Å)				
U - O	2.154	2.171	2.253	2.166
U - C_{CO}	2.331	2.364	2.316	2.395
C_{CO} - O_{CO}	1.169	1.159	1.181	1.158
angles (°)				
O - U - C_{CO}	101.2	95.7	104.2	97.0
U - C_{CO} - O_{CO}	178.7	178.1	178.4	177.7
U - O - C	171.2	170.3	177.9	172.7

Table S3. Selected geometrical parameters comparison between the optimized structures at different spin states and the experimental one for complex **3**.

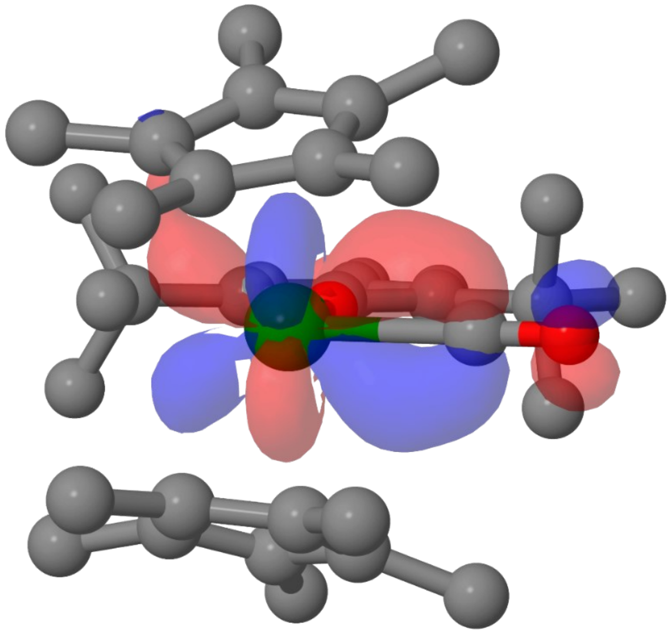

Figure S18. 3D representation of the SOMO-2 of complex **3** showing the major contribution to the backbonding in CO.

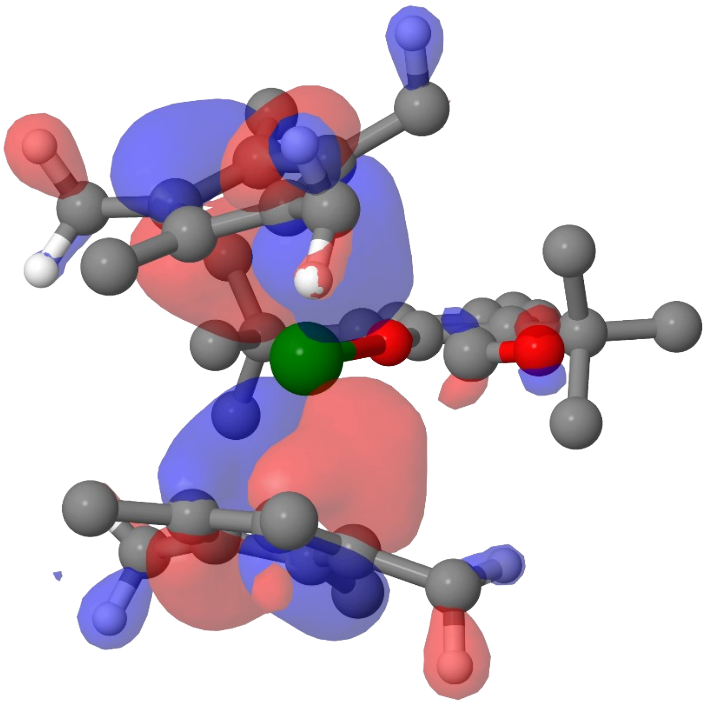

Figure S19. 3D representation of the HOMO-4 of complex **3** showing the interaction of the $(C_5Me_5)^{1-}$ ligand which has a minor contribution to the backbonding in CO.

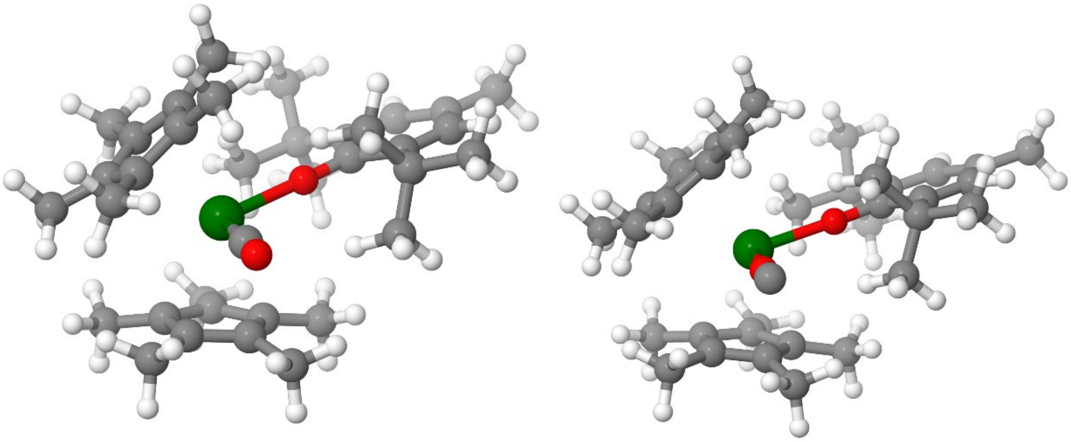

$\Delta_r H$ (kcal/mol)	0.0	+18.2
$\Delta_r G$ (kcal/mol)	0.0	+16.9

Figure S20. Energetic difference between carbon bound versus oxygen bound CO in complex **3**.

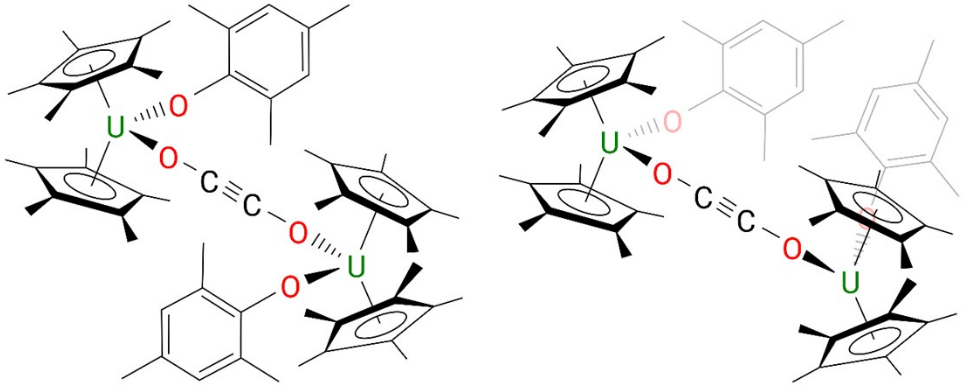

$\Delta_r H$ ($\Delta_r G$) in kcal.mol^{-1}	**0.0** **(0.0)**	**+0.4** **(+0.4)**

Figure S21. Effect of the aryloxide position to the stability of complex **4**.

References

1. J. C. Wedal, J. W. Ziller, F. Furche and W. J. Evans, *Inorg. Chem.*, 2022, **61**, 7365-7376.
2. R. J. Ward, D. Pividori, A. Carpentier, M. L. Tarlton, S. P. Kelley, L. Maron, K. Meyer and J. R. Walensky, *Organometallics*, 2021, **40**, 1411-1415.
3. J. B. Hendrickson and M. S. Hussoin, *J. Org. Chem.* 1989, **54**, 1144-1149.
4. S. Van Mileghem and W. M. De Borggraeve, *Org Process Res Dev*, 2017, **21**, 785-787.
5. Apex3, AXScale and SAINT, version 2017.3-0, Bruker AXS, Inc., Madison, WI, 2017.
6. G. M. Sheldrick., 2013, v.2013-2011.
7. G. Sheldrick, *Acta Crys. A*, 2015, **71**, 3-8.
8. G. Sheldrick, *Acta Crys. C*, 2015, **71**, 3-8.
9. A. Thorn, B. Dittrich and G. M. Sheldrick, *Acta Crystallogr. Sect. A: Crystallogr.*, 2012, **68**, 448 - 451.
10. A. L. Spek, PLATON: A Multipurpose Crystallographic Tool, 2001, v. 140621.
11. M. J. Frisch, G. W. Trucks, H. B. Schlegel, G. E. Scuseria, M. A. Robb, J. R. Cheeseman, G. Scalmani, V. Barone, G. A. Petersson, H. Nakatsuji, X. Li, M. Caricato, A. V. Marenich, J. Bloino, B. G. Janesko, R. Gomperts, B. Mennucci, H. P. Hratchian, J. V. Ortiz, A. F. Izmaylov, J. L. Sonnenberg, Williams, F. Ding, F. Lipparini, F. Egidi, J. Goings, B. Peng, A. Petrone, T. Henderson, D. Ranasinghe, V. G. Zakrzewski, J. Gao, N. Rega, G. Zheng, W. Liang, M. Hada, M. Ehara, K. Toyota, R. Fukuda, J. Hasegawa, M. Ishida, T. Nakajima, Y. Honda, O. Kitao, H. Nakai, T. Vreven, K. Throssell, J. A. Montgomery Jr., J. E. Peralta, F. Ogliaro, M. J. Bearpark, J. J. Heyd, E. N. Brothers, K. N. Kudin, V. N. Staroverov, T. A. Keith, R. Kobayashi, J. Normand, K. Raghavachari, A. P. Rendell, J. C. Burant, S. S. Iyengar, J. Tomasi, M. Cossi, J. M. Millam, M. Klene, C. Adamo, R. Cammi, J. W. Ochterski, R. L. Martin, K. Morokuma, O. Farkas, J. B. Foresman and D. J. Fox, *Journal*, 2016.
12. J. P. Perdew, J. A. Chevary, S. H. Vosko, K. A. Jackson, M. R. Pederson, D. J. Singh and C. Fiolhais, *Phys. Rev. B*, 1992, **46**, 6671-6687.
13. A. D. Becke, *J. Chem. Phys.*, 1993, **98**, 5648-5652.
14. X. Cao and M. Dolg, J MOL STRUC-THEOCHEM, 2004, **673**, 203-209.
15. X. Cao, M. Dolg and H. Stoll, *The Journal of Chemical Physics*, 2003, **118**, 487-496.
16. R. Ditchfield, W. J. Hehre and J. A. Pople, *J. Chem. Phys.*, 1971, **54**, 724-728.
17. W. J. Hehre, R. Ditchfield and J. A. Pople, *J. Chem. Phys.*, 1972, **56**, 2257-2261.
18. P. C. Hariharan and J. A. Pople, *Theor. Chim. Acta*, 1973, **28**, 213-222.
19. A. E. Reed, L. A. Curtiss and F. Weinhold, *Chem. Rev.*, 1988, **88**, 899-926.
